# Vasicine Attenuates Allergic Asthma by Suppressing Mast Cell Degranulation and Th2 Inflammation via Modulation of the FcεRI/Lyn + Syk/MAPK Pathway

**DOI:** 10.3390/ph19010190

**Published:** 2026-01-22

**Authors:** Lu Qu, Wenxia Du, Zizai Ren, Mengmeng Chen, Xiangnong Wu, Xue Cao, Gaoxiong Rao, Xiaoyun Tong, Feng Huang, Yun Sun

**Affiliations:** 1 Department of Chinese Medicine, Yunnan University of Chinese Medicine, Kunming 650500, China; qululuhan88@163.com (L.Q.); dwx950529@163.com (W.D.); renzizai98@163.com (Z.R.); cm18285396380@163.com (M.C.); 89013@ynucm.edu.cn (G.R.); xiaoyuntong_ynutcm@163.com (X.T.); 2 School of Public Health, Kunming Medical University, Kunming 650500, China; 3 The First Afffliated Hospital of Yunnan University of Chinese Medicine, Kunming 650500, China; wxn7321@126.com; 4 Department of Laboratory Animal Science, Kunming Medical University, Kunming 650500, China; dir1865@163.com; 5 Yunnan Key Laboratory of Southern Medicinal Utilization, Yunnan University of Chinese Medicine, Kunming 650500, China

**Keywords:** vasicine, allergic asthma, inflammation, metabolomics, molecular docking, mast cells, FcεRI/Lyn + Syk/MAPK

## Abstract

**Background**: Vasicine (Vas) is a quinazoline alkaloid derived from Adhatoda vasica Nees, which has good anti-allergic asthma and anti-inflammatory effects. However, its specific functional mechanism on allergic asthma is unclear. This study aims to investigate the protective effect of Vas on allergic asthma and its underlying mechanisms. **Methods**: Initially, the therapeutic effects of Vas were assessed in ovalbumin-sensitized BALB/c mice using airway hyperresponsiveness (AHR), histopathological examinations, immunohistochemistry, and enzyme-linked immunosorbent assays (ELISA). Subsequently, a non-targeted metabolomic analysis was performed to examine the influence of Vas on lung metabolites, while molecular docking was utilized to clarify the mechanisms by which Vas intervenes in allergic asthma. Lastly, RBL-2H3 cells were employed in vitro to validate the metabolomic findings by measuring intracellular Ca^2+^ concentrations, in addition to conducting ELISA and Western blot analyses. **Results**: In vivo, Vas alleviates AHR in mice with allergic asthma, enhances histopathological conditions, and reduces inflammatory factors. Non-targeted metabolomics analyses indicate that the primary pathway implicated in its intervention in allergic asthma may be the FcεRI pathway. Furthermore, molecular docking techniques were utilized to evaluate the binding affinity between Vas and proteins associated with this pathway. In vitro, Vas effectively inhibits degranulation in RBL-2H3 cells and diminishes the release of inflammatory factors by modulating the FcεRI/Lyn + Syk/MAPK pathway. **Conclusions**: These findings indicate that Vas may effectively alleviate allergic asthma by reducing inflammatory responses, decreasing AHR, and improving histopathological features. Furthermore, Vas seems to inhibit mast cell degranulation and modulate the FcεRI/Lyn + Syk/MAPK pathway.

## 1. Introduction

Allergic asthma is a longstanding condition characterized by recurring episodes of significant respiratory difficulty, which are referred to as exacerbations of allergic asthma. These exacerbations lead to approximately 1.8 million hospital admissions each year, highlighting the severity and impact of this chronic airway disease on public health [1]. The underlying mechanism contributing to allergic asthma is primarily airway inflammation, which plays an essential role in both the onset and progression of the disease [2]. This inflammatory process can have profound negative effects on respiratory function, underscoring the need for effective management and treatment strategies. Key players in the inflammatory response associated with allergic asthma include a variety of immune cell types, with mast cells (MCs), eosinophils (EOSs), and T lymphocytes being particularly prominent [2]. These cells contribute to the pathological changes observed in the airways of individuals suffering from this condition. Furthermore, a diverse array of cytokines, including interleukin IL-4, IL-5, IL-9, and IL-13 [3], operate within a sophisticated network that exacerbates airway responsiveness. This network not only heightens the sensitivity of the airways but also increases mucus production, leads to the leakage of blood vessels, and results in the constriction of the airways [4]. Collectively, these processes characterize the complex and multifaceted nature of allergic asthma, emphasizing the importance of targeting such pathways in therapeutic interventions.

The interaction among MCs, type 2 helper T cells (Th2), and allergic asthma represents a critical immunological pathway involved in the manifestation of allergic asthma [5]. In this context, inhaled allergens are initially captured and processed by antigen-presenting cells, specifically dendritic cells, which then present these antigens to naive T cells. This presentation instructs naive T cells to differentiate into Th2 cells, which are pivotal in orchestrating the immune response [6]. Th2 cells contribute to the creation of a distinct type 2 immune microenvironment through the secretion of specific cytokines, such as IL-4, IL-5, etc. Additionally, these cytokines stimulate the production of immunoglobulin E (IgE), a key player in allergic responses [7]. Once produced, IgE influences MCs by sensitizing and activating them. This activation leads to the release of a variety of inflammatory mediators that provoke the pathophysiological alterations associated with asthma [8]. Moreover, activated MCs play a crucial role in perpetuating and amplifying the Th2 immune response. Upon subsequent exposure to the same allergen, the allergen binds to IgE antibodies that are attached to the surface of MCs. This interaction activates the high-affinity receptor FcεRI, which triggers a complex series of intracellular signaling events [8]. Important kinases such as Lyn, Syk, and MAPKs are involved in this signaling cascade [9]. The activation process begins with Lyn, which, upon FcεRI activation, becomes activated and induces the phosphorylation of immunoreceptor tyrosine-based activation motifs (ITAMs). This phosphorylation facilitates the recruitment and activation of Syk kinase [10]. The downstream signaling pathways activated during this cascade include MAPK, calcium ions, and NF-κB, culminating in the degranulation of MCs. Degranulation is characterized by the release of preformed mediators, such as histamine and tryptase, in addition to the synthesis of newly formed lipid mediators, like leukotrienes and prostaglandins. This cascade further leads to the production of various cytokines and chemokines, effectively triggering allergic responses once more [2].

**Figure 1:**
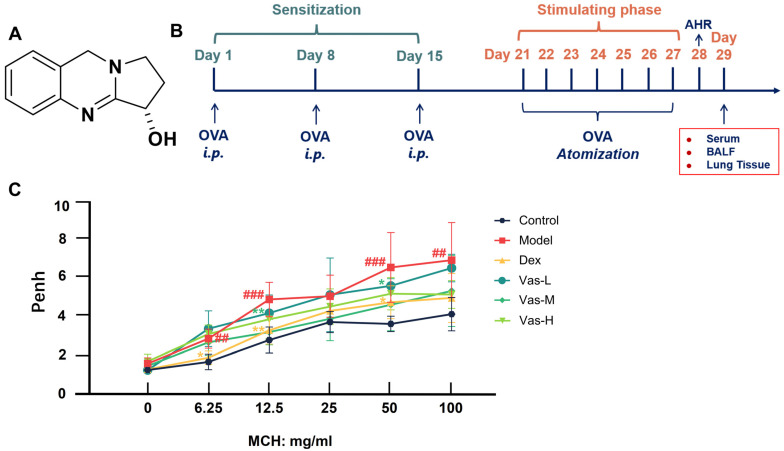

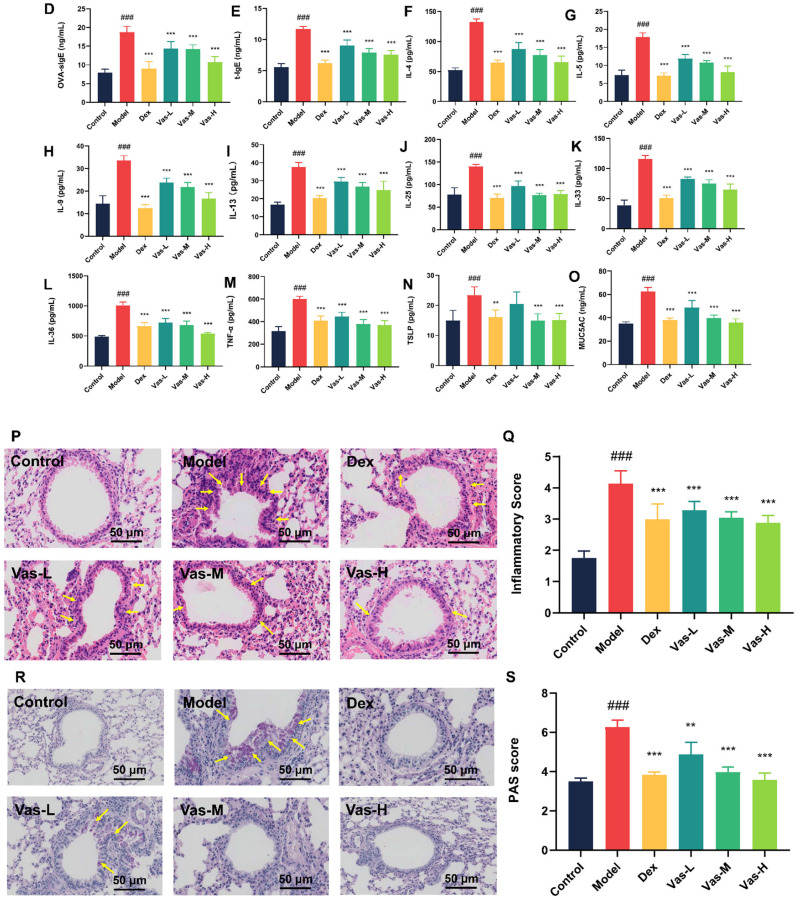

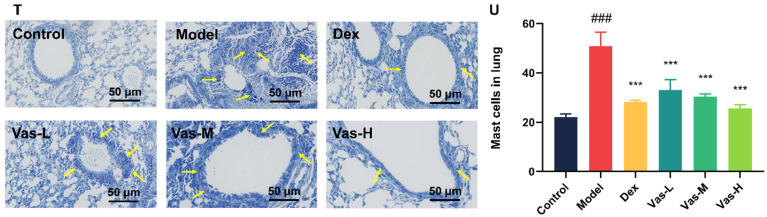
Beneficial impact of Vas in mitigating asthma symptoms in OVA-induced mice. Chemical structure of Vas (**A**). Schematic illustration of the mouse model for OVA-induced asthma (**B**). The influence of Vas on AHR in asthmatic mice (**C**). [AHR assessment in asthmatic mice was conducted via the MCH stimulation test following 28 days of specified treatment]. Serum concentrations of OVA-sIgE (**D**) and t-IgE (**E**). BALF levels of IL-4 (**F**), IL-5 (**G**), IL-9 (**H**), IL-13 (**I**), IL-25 (**J**), IL-33 (**K**), IL-36 (**L**), TNF-α (**M**), TSLP (**N**), and MUC5AC (**O**). Representative lung sections embedded in paraffin and stained with H&E (magnification, ×200) (**P**). Inflammatory scoring based on H&E staining (**Q**). Lung tissue sections subjected to PAS staining (magnification, ×200) (**R**). The PAS scoring (**S**). Lung sections stained utilizing the TB method (magnification, ×200) (**T**). Count of MCs present in the lung tissue (magnification, ×200) (**U**). The yellow arrows highlighted the areas of lesions. Data are illustrated as means ± SEM (*n* = 6). ## *p* < 0.01, ### *p* < 0.001, in comparison to the control. * *p* < 0.05, ** *p* < 0.01 and *** *p* < 0.001, relative to the model. Dexamethasone acetate, Dex (1 mg/kg); Vasicine, Vas; Vas-L (25 mg/kg); Vas-M (50 mg/kg); Vas-H (100 mg/kg).

*Adhatoda vasica* is a perennial herb that has been utilized for over 2000 years in Ayurvedic and Unani medical systems to treat respiratory disorders [11]. Vasicine (Vas), the principal quinazoline alkaloid derived from *A. vasica* (Figure 1A), has been demonstrated to induce bronchodilation and tracheal muscle relaxation at lower concentrations, while exhibiting significant protective effects against histamine-induced bronchospasm in guinea pigs at higher concentrations [11,12,13,14]. Literature studies indicate that the vasicine analog R8 can inhibit the differentiation of T cells into Th2 cells by interfering with the binding of IL-4 to its receptor. This effect subsequently reduces the phosphorylation level of STAT6 and the expression of GATA3 in asthmatic mouse models, thereby contributing to the intervention in asthma [15]. Previous studies conducted by our research group revealed that Vas can alleviate atopic dermatitis by inhibiting the infiltration of skin mast cells, reducing serum IgE levels, and suppressing the expression of cytokines such as IL-4, IL-5, IL-9, and IL-13 in both skin tissues and serum [16]. Literature indicates that atopic dermatitis, allergic rhinitis, and allergic asthma are collectively referred to as the ‘atopic triad’ or ‘atopic march,’ as they are all classified as type 2 inflammatory diseases [17]. Cytokines such as IgE, IL-4, IL-5 etc are closely associated with MCs [2]. Consequently, the research team proposed the hypothesis that Vas alleviates allergic asthma by inhibiting MC-mediated airway allergic inflammation, which was validated through in vivo and in vitro experiments. The results demonstrated that Vas effectively mitigates allergic asthma by reducing inflammatory responses, decreasing airway hyperresponsiveness (AHR), and improving histopathological features. Furthermore, Vas was found to inhibit MC degranulation and regulate the FcεRI/Lyn/Syk/MAPK signaling pathway. These results offer a theoretical foundation for utilizing Vas in the management of allergic conditions.

## 2. Results

### 2.1. Effect of Vas on AHR in OVA-Challenged Mice

The modeling process of this experiment is shown in Figure 1B. Airway constriction that is excessive serves as a major clinical characteristic of allergic asthma. Sensitization and subsequent challenge with OVA lead to the development of AHR in mice [18]. Results from experiments demonstrated that as methacholine (MCH) concentrations increased, the Penh values across all mouse groups displayed a trend of gradual increase, with the model group exhibiting a more pronounced rise in comparison to the other groups (Figure 1C). In contrast to the model group, the Penh values in the Vas groups reduced following exposure to various concentrations of MCH. Notably, the alterations seen in the Dex were akin to those observed in the Vas-H (Figure 1C). Considering the multifaceted validation of the results, we have included the Area Under the Curve (AUC) findings in Supplementary Material S1.

### 2.2. Effect of Vas on Serum and BALF in OVA-Challenged Mice

Serum levels of OVA-sIgE and t-IgE were also assessed. The findings indicated that the concentrations of OVA-sIgE and t-IgE in the model were considerably elevated compared to the control (*p* < 0.01). Conversely, the levels observed in the Dex, Vas-H, Vas-M, and Vas-L were notably lower than those in the model (*p* < 0.01; Figure 1D,E). Compared to the model, Vas-H reduced the secretion levels of these two factors by 42.9% and 35.4%, respectively, whereas Dex decreased them by 52.1% and 46.8%.

Marked differences were observed in the concentrations of IL-4, IL-5, IL-9, IL-13, IL-25, IL-33, IL-36, TNF-α, and TSLP in BALF when comparing the model to the Vas. The levels of the aforementioned cytokines were considerably lower in the model compared to both the Vas-H and Vas-M (*p* < 0.001; Figure 1F,N). Compared to the model group, Vas-H reduced the secretion levels of the aforementioned cytokines by 50.3%, 54.1%, 50.3%, 34.2%, 43.9%, 44.0%, 46.8%, 38.7%, and 35.0%, respectively, while Dex reduced these levels by 48.6%, 60.0%, 62.9%, 46.0%, 49.8%, 56.0%, 34.7%, 32.4%, and 30.8%, respectively. The therapeutic effects observed in both the Vas-H and Dex groups were comparable.

In individuals suffering from allergic asthma, MUC5AC secretion in the airways is primarily produced by hyperplastic goblet cells. The amounts of MUC5AC have shown a positive correlation with the extent of inflammatory cell invasion in the airways. In comparative analyses, the model exhibited significantly elevated MUC5AC levels compared to the control, with statistical significance indicated at *p* < 0.001 (Figure 1O). Following the therapeutic intervention, a notable decrease in MUC5AC levels was observed across all treated groups. Specifically, reductions were noted in the Dex, Vas-H, Vas-M, and Vas-L groups, underscoring the efficacy of the treatment in mitigating MUC5AC production in these asthmatic mice. In comparison to the model, Vas-H resulted in a 42.9% reduction in the secretion level of MUC5AC, whereas Dex led to a decrease of 38.9%.

### 2.3. Effect of Vas on Lung Histopathological Changes in OVA-Challenged Mice

Lung tissue samples underwent hematoxylin-eosin (H&E) staining to assess the effects of Vas on lung histopathological changes. Mice that were subjected to OVA challenges exhibited substantial infiltration of inflammatory cells in the perivascular areas, accompanied by thickening of the airway epithelium. In contrast, the administration of either Dex or Vas was found to alleviate the pathological modifications induced by OVA, as illustrated in Figure 1P,Q. In addition, Periodic Acid Schiff (PAS) staining revealed a significant increase in PAS-positive scores in the model mice when compared to the control, as shown in Figure 1R,S. Following treatment interventions, there was a notable improvement in goblet cell proliferation and airway mucus secretion, particularly in the groups receiving Dex, Vas-H, Vas-M, and Vas-L, with the Vas-H group displaying the most remarkable enhancements. Furthermore, the presence of MCs in the lung tissue was evaluated through toluidine blue (TB) staining. The findings indicated a significant rise in MC infiltration in mice treated with OVA. Interestingly, mice treated with Vas exhibited a reduced level of MC infiltration in their lung tissue compared to those treated solely with OVA, as depicted in Figure 1T,U.

### 2.4. Effects of Vas on the Immunohistochemical Staining from the Lungs of Asthmatic Mice

**Figure 2:**
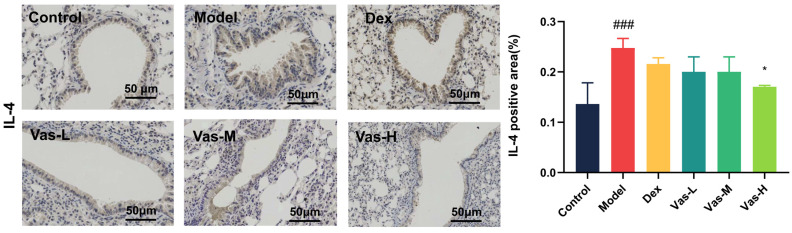

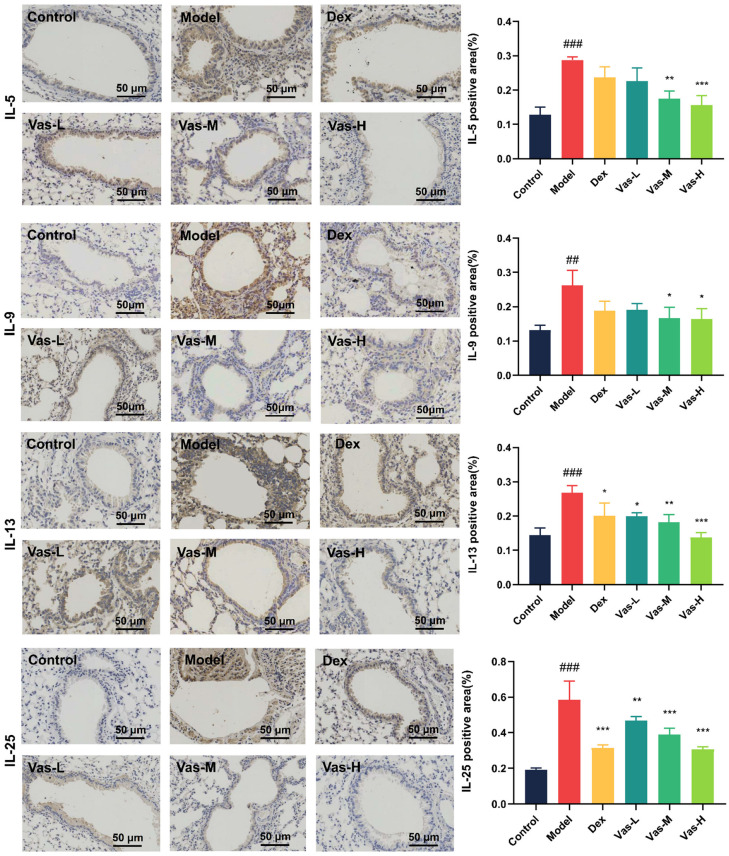

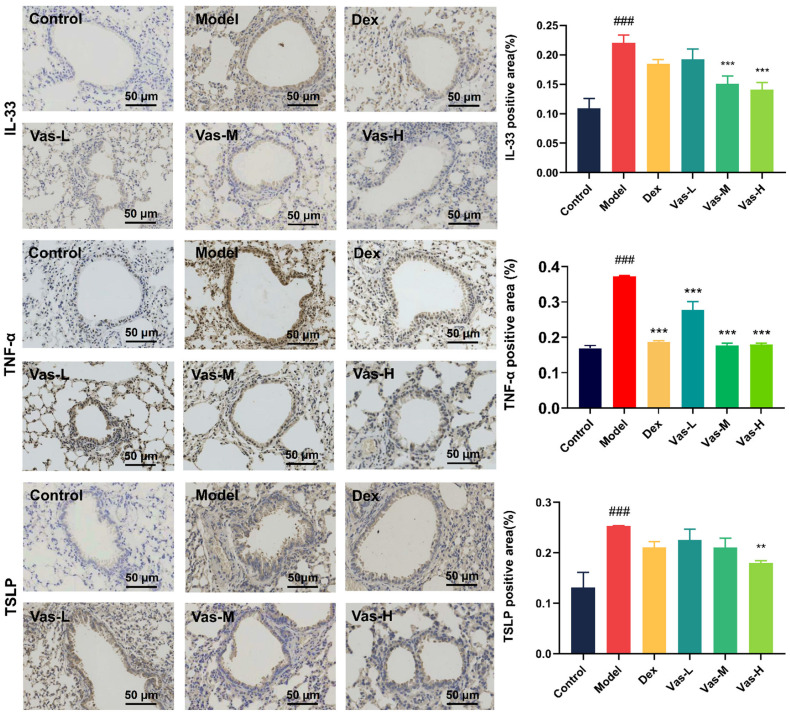
Immunohistochemical staining of IL-4, IL-5, IL-9, IL-13, IL-25, IL-33, TNF-α, and TSLP in the lung tissues (×200). Data are illustrated as means ± SEM (*n* = 6). ## *p* < 0.01, ### *p* < 0.001, compared with the control group. * *p* < 0.05, ** *p* < 0.01, and *** *p* < 0.001, compared with the model. Dexamethasone acetate, Dex (1 mg/kg); Vasicine, Vas; Vas-L (25 mg/kg); Vas-M (50 mg/kg); Vas-H (100 mg/kg).

Immunohistochemical analysis (Figure 2) indicated the upregulation of IL-4, IL-5, IL-9, IL-13, IL-25, IL-33, TNF-α, and TSLP in the lungs of model. Administration of Vas-H reduced the expressions of all these markers.

### 2.5. Screening and Identification of Potential Asthma Biomarkers in Mice

**Figure 3:**
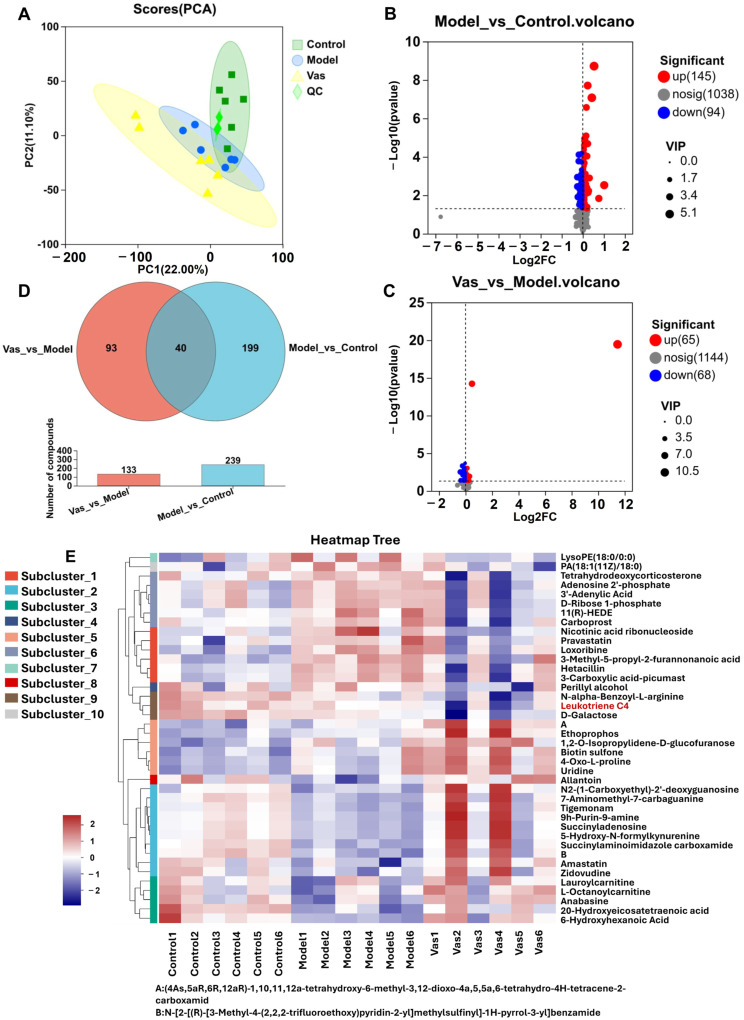

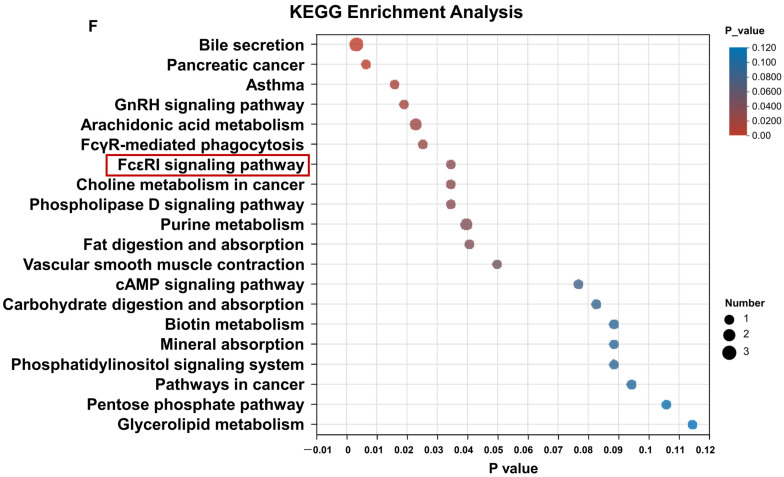
Lung tissue metabolomics study of asthma. PCA profile of each group of lung tissue samples (**A**). The volcano plot illustrates both upregulated and downregulated differential metabolites between the control and the model (**B**). The volcano plot shows the upregulated and downregulated differential metabolites when comparing the Vas to the model (**C**). An intersection diagram depicts the differential metabolites identified in each category of lung tissue samples (**D**). Heat map analysis highlights 40 distinct metabolites (VIP > 1 and *p* < 0.05) in the control, model and Vas. Blue indicates a reduction in the accumulation of these metabolites, while red signifies an increase (**E**). KEGG enrichment analysis (**F**). (Leukotriene C4 is highlighted in red font as our metabolite of interest, a critical mediator of allergic inflammation and airway hyperresponsiveness in asthma. The red box highlights the FcεRI signaling pathway, our primary pathway of interest, which drives mast cell activation and allergic inflammation central to asthma pathogenesis.)

Distinct variations were noted in the lung tissue samples from the control and model, as demonstrated by multivariate statistical techniques and PCA chart examination, highlighting notable differences in the endogenous metabolites between these groups (Figure 3A). The figures revealed some overlap between the model and Vas groups, indicating that the metabolites from the various treatment groups were quite similar prior to collection, although clear distinctions existed between the groups. In light of this observation, we advanced to the subsequent phase of differential metabolite analysis.

In the course of this experiment, researchers successfully identified a comprehensive total of 1021 metabolites when utilizing cation mode. This indicates a significant breadth of chemical compounds detectable in this particular mode of analysis. Additionally, the study revealed an even greater number of metabolites, amounting to 1695, in anion mode. The criteria for differential metabolite screening included *p* < 0.05 and VIP > 1, with results aggregated for both anions and cations. When the model was compared to the control, it was found that 145 metabolites were significantly upregulated, whereas 94 metabolites were significantly downregulated (Figure 3B,D). When comparing the Vas to the model, 65 metabolites showed significant upregulation, while 68 metabolites were significantly downregulated (Figure 3C,D). To delve deeper into the effects of Vas, we utilized these differential metabolites to discern the differences among the three groups for subsequent analysis.

Subsequent examination uncovered that 40 distinct metabolites were shared among the control, model, and Vas groups (Supplementary Material S2, Table S1), suggesting that Vas influences these metabolites to exert its effects. By analyzing the potential biomarkers, the structures of 20 endogenous metabolites were elucidated, primarily encompassing organic acids, lipids (such as phospholipids, fatty acids, and eicosanoids), carbohydrates, nucleic acids, peptides, vitamins and cofactors, steroids, hormones, neurotransmitters, and antibiotics. The heatmap analysis illustrating the relative content, depicted in Figure 3E, further highlights the biomarkers associated with asthma while revealing variations in the levels of each endogenous substance. The intensity of the colors on the heat map reflects the relative content, where red and blue denote low and high content, respectively. Darker colors signify more pronounced differences in relative content. Based on hierarchical cluster analysis of the heat map, the mice were categorized into control, model, and Vas groups.

A topological enrichment analysis was conducted on the previously recognized 40 potential biomarkers utilizing the KEGG database (Figure 3F). These biomarkers were primarily associated with asthma, metabolism of arachidonic acid, purine metabolism, and the FcεRI signaling pathway. Leukotrienes were identified as the principal differential metabolites that were enriched within the FcεRI signaling pathway. The FcεRI pathway serves as the ‘initiation command,’ while leukotriene acts as one of the ‘executive weapons.’ The activation of the FcεRI pathway directly enhances the synthesis and release of leukotrienes, representing the ‘initiating factor’ for their production. Once induced by the FcεRI pathway, leukotrienes become independent potent inflammatory signals that synergize with other mediators, such as histamine, to collectively amplify allergic inflammatory responses [19]. The FcεRI receptor, which responds to IgE, is found in high concentrations on the surfaces of MCs. Upon detection of an allergen, the rapid cross-linking of FcεRI on MC surface with multivalent antigens triggers an activation of MCs, resulting in the substantial release of allergic mediators.

### 2.6. Virtual Molecular Docking Verification

The findings from non-targeted metabolomics suggested that the mechanism underlying Vas intervention in asthma is associated with the FcεRI pathway. MCs serve as crucial effector cells in allergic responses mediated by FcεRI. To confirm interactions between Vas and critical proteins within the FcεRI signaling pathway—namely, Lyn, Syk, and MAPK (ERK1/2, JNK, P38)—virtual molecular docking was employed [20,21,22,23,24].

According to the data presented in Table 1, Vas exhibits relatively low binding free energy and docking scores with Lyn, Syk, p38, ERK1, and ERK2 proteins, indicating that the complexes formed between Vas and these five proteins possess high binding stability. However, although Vas shows a low docking score with the JNK protein, its binding free energy is relatively high, suggesting weaker binding stability between the two. Additionally, Vas demonstrates both high docking scores and binding free energy with the Syk protein, indicating the poorest interaction stability among them.

To further validate the binding affinity of Vas with various kinase targets, this study employed a unified molecular docking strategy to dock classical inhibitors (positive control compounds) [25,26,27,28,29] of each target protein with their corresponding targets, as shown in Table 1. The biological significance was analyzed as follows: For the Lyn target, a key kinase regulating immune cell signaling, the XP docking score (−8.486) and MM-GBSA binding free energy (−50.45 kcal/mol) of the positive control compound PP2 were significantly lower than those of Vas, which had a docking score of −5.012 and a binding free energy of −35.49 kcal/mol. This indicates that PP2 has superior binding stability to Lyn. However, Vas still exhibited relatively low docking scores and binding free energy, demonstrating its effective binding capability with Lyn, thus providing molecular-level evidence for its potential modulation of Lyn-mediated signaling pathways. For the Syk target, one of the core kinases in immune cell signal transduction, the positive control compound Sovleplenib showed a docking score of −3.198 and MM-GBSA binding free energy of −38.40 kcal/mol, both at relatively low levels, indicating stable binding with Syk. Similarly, Vas exhibited a low docking score of −4.463 and binding free energy of −29.84 kcal/mol, demonstrating favorable binding stability with Syk. In the ERK1 target, a core kinase of the MAPK pathway involved in cell proliferation and inflammatory responses, the positive control compound BVD-523 exhibited relatively weak binding stability, with a docking score of −4.944 and a binding free energy of −18.89 kcal/mol. In contrast, Vas demonstrated an even lower docking score of −4.043 and a significantly reduced binding free energy of −32.43 kcal/mol. The decrease in binding free energy typically indicates enhanced thermodynamic stability of the protein-ligand complex, suggesting that Vas has superior binding stability with ERK1 compared to BVD-523. This observation underscores Vas’s favorable binding activity towards ERK1 and supports its regulatory effects on ERK1-mediated cellular functions. For the ERK2 target, a MAPK family member that overlaps functionally with ERK1, BVD-523 exhibited a slightly higher docking score of −3.751 compared to Vas’s −4.400, along with a marginally lower binding free energy of −31.40 kcal/mol versus Vas’s −29.41 kcal/mol. Both compounds displayed relatively low docking scores and binding free energies, indicating that Vas possesses comparable binding stability with ERK2 to the positive control compound. This further corroborates its potential for broad-spectrum binding across the ERK kinase family. In the JNK target, a kinase that regulates apoptosis in the MAPK pathway, the positive control compound SP600125 exhibited poor binding stability, with a docking score of −2.660 and a binding free energy of −9.56 kcal/mol. Vas demonstrated a slightly lower docking score of −3.080; however, its binding free energy (−10.92 kcal/mol) remained relatively high, as binding free energies typically below −30 kcal/mol indicate stable binding. Both compounds exhibited unstable binding, consistently suggesting that JNK is not the preferential binding target for Vas. Subsequent experiments should prioritize investigating Vas’s regulatory effects on other targets. In the P38 target, a MAPK kinase involved in inflammatory and stress responses, the positive control compound SB4 displayed a lower docking score (−5.469) compared to Vas (−4.308), yet its binding free energy (−28.56 kcal/mol) was slightly higher than that of Vas (−32.81 kcal/mol). Both compounds exhibited relatively low docking scores and binding free energies, indicating comparable binding stability, although Vas demonstrated lower binding free energy. This difference suggests that the Vas-P38 complex possesses superior thermodynamic stability, further reinforcing its advantage in P38 binding capability. This study validated the binding characteristics of vasicine (Vas) with multiple kinase targets through molecular docking analysis: Vas exhibited favorable binding stability with Lyn, Syk, ERK1/2, and P38, demonstrating superior or comparable binding activity to ERK1 and P38 when compared with positive control compounds; however, it showed poor binding stability with JNK, indicating that JNK is not its predominant target.

**Figure 4:**
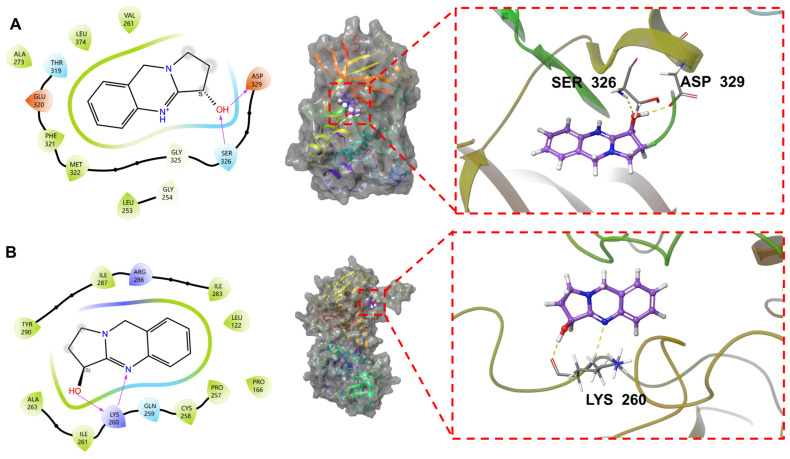

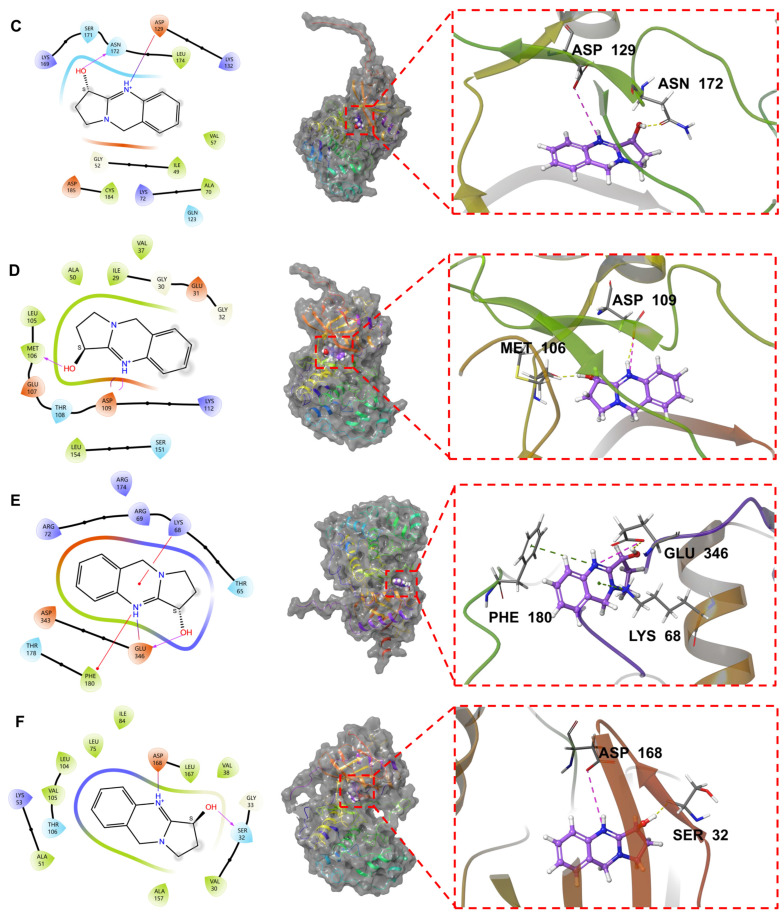
Two-dimensional and three-dimensional diagrams of docking between Vas and Lyn (**A**), Syk (**B**), ERK1 (**C**), ERK2 (**D**), JNK (**E**), and P38 (**F**) proteins. Note: Yellow represents hydrogen bonds, lavender represents salt bridges, and green represents π cation bonds.

To further elucidate the molecular mechanism underlying the stable binding of Vas with the Lyn, Syk, p38, ERK1, and ERK2 proteins, molecular docking analysis presented in Figure 4 demonstrates that Vas can effectively penetrate the active pocket of the Lyn protein. This interaction involves the formation of hydrophobic interactions with residues VAL261, LEU374, PHE321, and MET322, alongside the establishment of one hydrogen bond each with residues ASP329 and SER326, collectively contributing to the stability of the complex. In the case of Syk, upon embedding into its active pocket, Vas forms hydrophobic interactions with residues ILE283, LEU122, PRO166, PRO257, and CYS258, while establishing two hydrogen bonds with LYS260, thereby synergistically enhancing binding affinity. Within the active pocket of the p38 protein, Vas engages in hydrophobic interactions with residues LEU167, ALA157, and VAL30, forms one hydrogen bond with SER32, and establishes a salt bridge with ASP168, with multiple forces working cooperatively to strengthen binding stability. For the ERK1 protein, after entering its active pocket, Vas forms one hydrogen bond with residue ASN172 and establishes one salt bridge with ASP129, providing a structural basis for complex stability. Finally, within the active pocket of the ERK2 protein, Vas forms hydrophobic interactions with residues ILE29, ALA50, LEU105, and MET106, while establishing one hydrogen bond with MET106, as well as one hydrogen bond and one salt bridge with ASP109, achieving stable binding through synergistic hydrophobic and polar interactions. (The specific binding modes, including hydrogen bonds and hydrophobic interactions, between each target protein and its corresponding positive control compound are detailed in Supplementary Material S5).

### 2.7. Effect of Vas on the Release of Degranulated β-HEX and Histamine from Antigen-Induced of RBL-2H3 Cells

**Figure 5:**
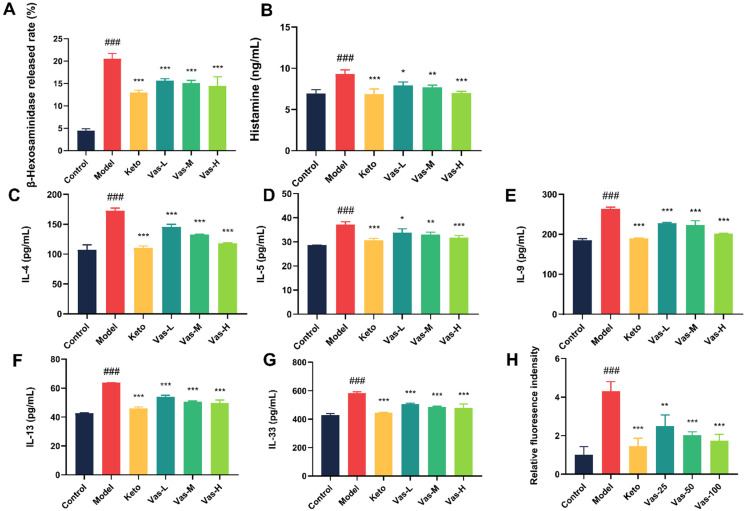

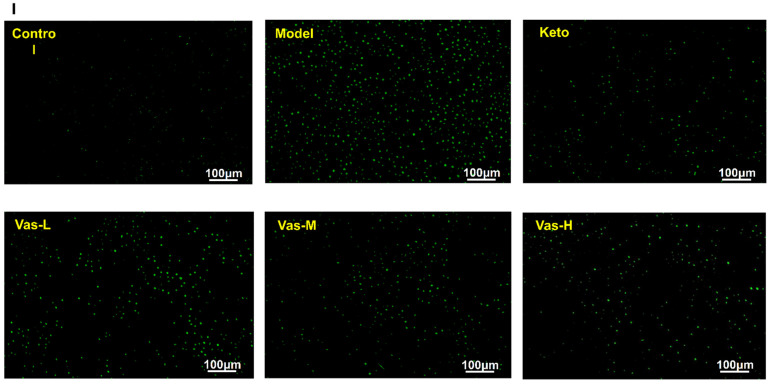
Influence of Vas on MCs. The impact of Vas on β-HEX induced by IgE (**A**), histamine (**B**), IL-4 (**C**), IL-5 (**D**), IL-9 (**E**), IL-13 (**F**), and IL-33 (**G**) production from RBL-2H3 cells. The impact of Vas on intracellular Ca^2+^ concentrations in IgE-induced activation of RBL-2H3 cells (×100) (**H**,**I**). The results from three independent experiments performed in triplicate were expressed as means ± SEM (*n* = 3). ### *p* < 0.001, compared with the control group. * *p* < 0.05, ** *p* < 0.01, and *** *p* < 0.001, compared with the model. Ketotifen, Keto (60 µM); Vasicine, Vas; Vas-L (25 µM); Vas-M (50 µM); Vas-H (100 µM).

In our study, we examined how Vas affects the degranulation process in antigen-stimulated RBL-2H3 cells to explore its potential anti-allergic properties [30]. We selected β-HEX and histamine release as a marker for degranulation [31]. The results indicated that Vas significantly inhibited β-HEX release during cell degranulation (*p* < 0.001; Figure 5A). Upon stimulation with antigens, RBL-2H3 cells release histamine. The histamine receptor antagonist, keto (Ketotifen Fumarate), effectively reduces this release. Additionally, Vas-H also exhibits the capability to inhibit histamine release, paralleling the effects observed with keto (Figure 5B). These findings imply that Vas plays a role in preventing the activation of MCs, thus leading to a decrease in β-HEX and histamine release.

### 2.8. Effect of Vas on the Release of Th2 Cytokines from RBL-2H3 Activation Degranulation

The release of essential Th2 factors, including IL-4, IL-5, IL-9, IL-13, and IL-33 from RBL-2H3 cells, was assessed using ELISA. In the model, the expression levels of these cytokines were markedly elevated in the supernatant of RBL-2H3 cells compared to those observed in the control. (*p* < 0.001; Figure 5C,G). Following the treatment with Keto and Vas, the expression levels of Keto and Vas were notably reduced compared to those in the model (*p* < 0.001), similar to the findings in the control group. Vas showed inhibitory effects, in which Vas-H was the most obvious, followed by Vas-M and Vas-L, which also indicated that Vas could also interfere with the disease by inhibiting the expressions of Th2 cytokines in MCs.

### 2.9. Effect of Vas on Intracellular Ca^2+^ Concentration in IgE-Induced Activation of RBL-2H3 Cells

The concentration of intracellular calcium ions (Ca^2+^) significantly influences the degranulation process of MCs, as highlighted in earlier research [32]. In our research, we specifically investigated the impact of Vas on intracellular Ca^2+^ levels within RBL-2H3 cells that were stimulated by DNP-IgE. Our findings revealed that, upon the challenge with DNP-IgE, there was a remarkable increase in intracellular Ca^2+^ levels, showing an up to four-fold elevation in the stimulated cells when compared to baseline levels in normal cells. This significant rise in Ca^2+^ concentration was effectively attenuated by the presence of Vas, as illustrated in Figure 5H,I. Additionally, we measured the inhibitory impacts of varying concentrations of Vas on the increase in intracellular Ca^2+^. The results indicated varying degrees of inhibition: Vas-L demonstrated a 42% reduction, Vas-M achieved a 53% reduction, and Vas-H resulted in a 60% reduction in the Ca^2+^ increase caused by the DNP-IgE stimulation. These findings indicate that Vas’s capability to suppress degranulation in RBL-2H3 cells mediated by DNP-IgE is probably associated with its ability to reduce the elevated intracellular Ca^2+^ concentrations during this process.

### 2.10. Effects of Vas on the Phosphorylation of Lyn, Syk, and MAPK in IgE-Stimulated RBL-2H3 Cells

**Figure 6:**
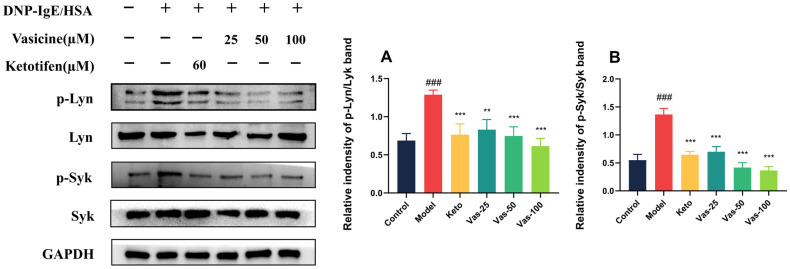

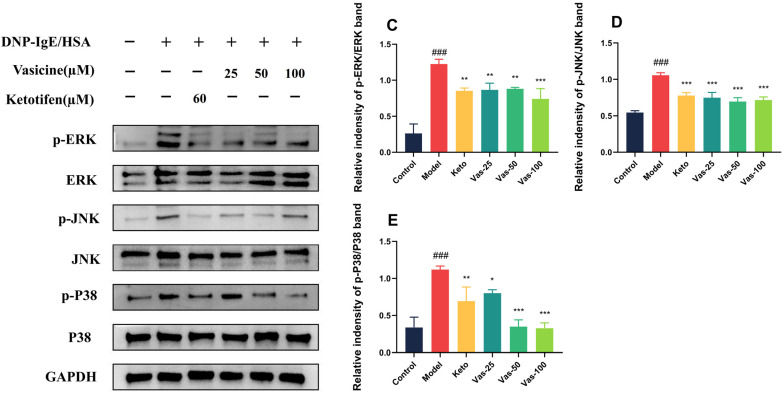
The influence of Vas on the expression of p-Lyn/Lyn (**A**), p-Syk/Syk (**B**), and MAPK [p-ERK/ERK (**C**), p-JNK/JNK (**D**), and p-P38/P38 (**E**)] proteins induced by IgE in RBL-2H3 cells was studied. Data obtained from three separate experiments, each conducted in triplicate, are presented as means ± SEM. The statistical significance was determined with ### *p* < 0.001 when compared to the control. Additionally, statistical significance was noted with * *p* < 0.05, ** *p* < 0.01, and *** *p* < 0.001 in comparison to the model. Ketotifen (Keto) was used at a concentration of 60 µM; Vasicine (Vas) was tested at doses of Vas-25 (25 µM), Vas-50 (50 µM), and Vas-100 (100 µM).

Western blotting was performed using RBL-2H3 cells to confirm the molecular docking results. Vas played a significant role in diminishing the activated degranulation of RBL-2H3 cells. This effect was achieved through the inhibition of several crucial proteins that are integral to the signaling pathway associated with IgE and FcεRI. Specifically, Vas interfered with the expression of proteins such as phosphorylated Lyn (p-Lyn), Lyn, phosphorylated Syk (p-Syk), and Syk. These proteins are essential components that operate upstream in the signaling cascade, and their reduced expression suggests a potential mechanism through which Vas exerts its inhibitory influence on cell activation and subsequent degranulation processes. (Figure 6A,B).

MAPK play a crucial role in modulating the rate of MC degranulation by influencing the IgE/FcεRI signaling pathway. These kinases are vital components of the downstream signaling cascades that are triggered following the activation of the p-Lyn/Lyn and p-Syk/Syk pathways. To further investigate the effects of Vas on these signaling mechanisms, we explored how Vas impacts the expression of MAPKs. Our findings revealed a significant elevation in the expression levels of MAPKs following stimulation with IgE in the experimental model. Conversely, treatment with Vas resulted in a notable decline in MAPK expression, especially in the high-dose subgroup, which exhibited a pronounced reduction in MAPK levels (see Figure 6C–E). Additionally, the results from the Western blot analysis supported the outcomes of the molecular docking study, reinforcing the conclusions drawn from our investigation.

## 3. Discussion

The findings from this study reveal that Vas treatment holds significant promise in suppressing mast cell-mediated allergic airway inflammation. To substantiate this hypothesis, we applied an OVA-induced allergic asthma model alongside IgE-stimulated RBL-2H3 cells to thoroughly assess the effects of Vas. In the context of our in vivo studies, the OVA-induced asthma model served as a foundational experimental framework, recognized as a traditional approach for investigating allergic asthma (Figure 1B) [33]. Allergic asthma is characterized by a distinct systemic immune response that features heightened levels of IgE as well as allergen-specific IgE, a result of B cell activation [7]. Our results indicate that Vas effectively dampened the systemic immune response observed in the OVA-induced asthma model, leading to a significant reduction in the serum levels of both OVA-sIgE and t-IgE (Figure 1D,E). In response to subsequent exposure to allergens, the inflammatory reaction within the tissues is primarily driven by the recruitment and infiltration of immune cells, culminating in the enhanced release of various mediators such as histamine, chemokines, and inflammatory cytokines [34]. The data illustrate that Vas not only mitigated mast cell infiltration within the lung tissues of the OVA-induced asthma model (Figure 1T,U) but also effectively suppressed the release of type 2 inflammatory cytokines, which include IL-4, IL-5, IL-9, and IL-13, in both BALF and lung tissues (Figure 1F–I and Figure 2). Furthermore, the study underscores the importance of interleukins IL-25, IL-33, and TSLP, as they function as upstream mediators of Th2 cytokines and are intricately associated with the pathogenesis of allergic asthma [35,36]. Notably, our findings demonstrate that Vas can inhibit the release of these pivotal mediators IL-25, IL-33, and TSLP within both BALF and lung tissues (Figure 1J,K,N and Figure 2). An additional key cytokine is IL-36. Belonging to the IL-1 superfamily, IL-36 is involved not only in inflammatory responses but is also produced by lung epithelial cells in response to stress, such as viral or bacterial infections and stimulation from house dust mites [37]. In BALF, the Vas-H group exhibited a more pronounced decrease in IL-36 secretion when compared to the model group and the Dex group (Figure 1L). This result suggests that Vas may inhibit the secretion of IL-36 by modulating its pathway, thus contributing to anti-inflammatory effects. Such findings will be a crucial area for future investigation.

Allergic asthma is clinically characterized by AHR, reversible airway obstruction, airway inflammation, and excessive mucus secretion [18]. AHR serves as a critical indicator for assessing respiratory function in patients with allergic asthma. The Pench parameter, an important metric for evaluating AHR, reflects the degree of airway resistance [38,39]. Research indicates that Vas can reduce airway resistance and improve AHR in mice with allergic asthma (Figure 1C). Patients with allergic asthma exhibit elevated levels of mucins, particularly MUC5AC, in the airways, alongside epithelial damage leading to ciliated cell shedding, significant goblet cell hyperplasia, and submucosal gland hypertrophy. Collectively, these pathological changes contribute to in-creased airway mucus secretion [40]. This experiment revealed that the model group exhibited pathological features in lung tissue, including extensive inflammatory cell infiltration and significant collagen deposition (Figure 1P–U). Following Vas treatment, inflammatory cell infiltration was reduced, and symptoms of airway obstruction were alleviated (Figure 1P–U). Vas was found to decrease MUC5AC levels in BALF, exhibiting a slightly greater reduction compared to Dex. This observation underscores the significant therapeutic effects of Vas on mice with allergic asthma. (Figure 1O).

**Figure 7:**
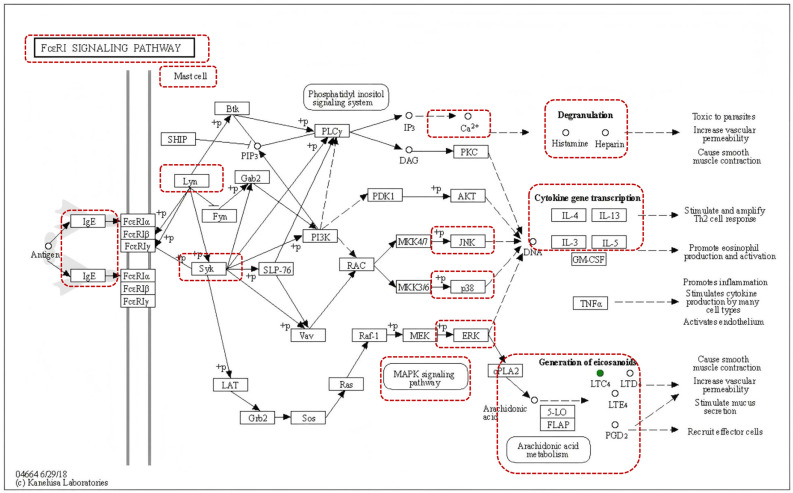
Schematic of the FcεRI signaling pathway (map04664) in mast cells, highlighting key kinase targets (Lyn, Syk, MAPK) involved in downstream processes (degranulation, cytokine transcription, eicosanoid generation). (Arrow conventions are represented as follows: solid arrows indicate direct protein–protein interactions or enzymatic reactions, while dashed arrows represent indirect signaling events or downstream functional outcomes. Arrows marked with “+p” signify phosphorylation events that activate downstream signaling molecules.The red boxes highlight key experimental targets, which include core signaling molecules (e.g., Lyn, Syk, and components of the MAPK pathway), critical second messengers (e.g., Ca^2+^), and functional endpoints (e.g., degranulation, cytokine gene transcription, and arachidonic acid metabolism) that are the focus of the present study.).

Previous pharmacological intervention studies utilizing the OVA model have primarily concentrated on cytokine levels and inflammatory cell counts. However, recent research has revealed that significant metabolic alterations also occur in patients with allergic asthma [41,42]. The detection of these metabolic changes can further elucidate the mechanisms underlying drug interventions for asthma [4,43]. An analysis of overlapping metabolites among the normal group, model group, and Vas group indicated that leukotrienes are associated with asthma, arachidonic acid, and the FcεRI pathway (Table S1 in Supplementary Material S2, Figure 3E). Furthermore, KEGG pathway enrichment analysis demonstrated that asthma, arachidonic acid, and the FcεRI pathway may represent key targets of Vas action. The FcεRI signaling pathway functions as the principal initiating pathway for critical cells (MCs) in allergic asthma and exhibits significant cross-regulation with the arachidonic acid metabolic pathway (Figure 7, map04664). When allergens such as OVA bind to the IgE-FcεRI complex on the surface of MCs and basophils, they initiate intracellular kinase cascades, including Syk and PLCγ, resulting in calcium influx and MAPK activation. This calcium influx subsequently triggers MC degranulation, leading to the release of substances such as histamine. Upon activation, MAPK triggers several simultaneous responses. First, it releases Th2-related inflammatory cytokines, including IL-4, IL-5, and IL-13, which further stimulate Th2 cells and induce a Th2 response. Second, the activation of MAPK, particularly through ERK phosphorylation, initiates the arachidonic acid cascade. Growing evidence indicates that ERK phosphorylation plays a critical regulatory role in the synthesis of LTC4 within the FcεRI signaling pathway [44,45,46,47]. This cascade ultimately activates the 5-lipoxygenase pathway, which catalyzes the production of potent inflammatory mediators—cysteinyl leukotrienes—from arachidonic acid [10]. The signaling cascade involving the Lyn/Syk/MAPK pathway, which is initiated by the cross-linking of FcεRI by antigen-IgE complexes, serves as the primary mechanism for mast cell degranulation and the subsequent release of pro-inflammatory mediators, including histamine and leukotrienes. Additionally, this cascade functions as an upstream regulator of arachidonic acid metabolism. This study highlights the significant alterations in pulmonary leukotriene levels following the administration of Vas. Furthermore, the enrichment of the FcεRI pathway identified through KEGG analysis indicates that the inhibitory effect of Vas on the FcεRI/MAPK pathway likely underpins its regulatory influence on the arachidonic acid metabolic pathway. These findings suggest that the therapeutic target of Vas may be closely associated with this pathway. Consequently, we further employed molecular docking technology to investigate the binding interactions between Vas and key proteins in the FcεRI signaling pathway.

The results of molecular docking and binding mode analysis jointly confirm that Vas can target Lyn, Syk, p38, and ERK family kinases, demonstrating distinct target specificity in binding activity. It exhibits superior binding stability with ERK1 and p38 compared to the positive control inhibitors, shows comparable binding activity to Lyn and ERK2 as the positive control, and displays no significant binding capacity to JNK. From a molecular mechanisms perspective, Vas stably embeds into the active pockets of target proteins, forming hydrophobic interactions with specific amino acid residues while synergizing with polar interactions such as hydrogen bonds and salt bridges to establish stable protein-ligand complexes. The binding residues and interaction types vary across different targets, with this structural specificity being the core determinant of differential binding stability among targets. These findings not only elucidate the binding characteristics of Vas with immune-inflammatory-related kinase targets at the molecular level but also identify its predominant target sites. This provides crucial theoretical foundations and target directions for further investigation into Vas’s regulation of kinase signaling pathways and its biological functions in intervening allergic asthma. (Table 1, Figure 4).

To further validate the aforementioned findings, this study conducted in vitro experiments using RBL-2H3 cells, which are widely utilized in IgE-mediated degranulation studies due to their high surface expression of IgE receptors and their structural and functional similarities to MC [30,48]. The in vitro results demonstrated that Vas effectively inhibited allergic responses, as evidenced by significantly reduced β-hexosaminidase and histamine release in activated RBL-2H3 cells (Figure 5A,B). Further investigation revealed that Vas suppresses MC degranulation by inhibiting Ca^2+^ influx and blocking the FcεRI signaling pathway (Figure 5H,I). Western blot analysis indicated that Vas could inhibit the phosphorylation of Lyn, Syk, and MAPK. A comparison with molecular docking results revealed an intriguing phenomenon: although Vas exhibited weaker binding energy with JNK, it still effectively suppressed their phosphorylation. This seemingly paradoxical finding suggests that Vas may not inhibit all targets equally but rather exerts its effects by targeting key up-stream nodes, such as Lyn kinase, within the signaling pathway, thereby generating a cascade of inhibitory effects. In the FcεRI signaling pathway, Lyn kinase acts as the ‘initiation switch’ for signal transduction. When FcεRI is cross-linked, Lyn, one of the earliest recruited and activated Src family kinases, is primarily responsible for phos-phorylating the ITAMs on the FcεRI β and γ chains [10]. Therefore, we speculate that the potent binding and inhibition of the early-stage kinase Lyn is the key mecha-nism by which Vas exerts its broad-spectrum inhibitory effects.

However, several limitations must be acknowledged. Firstly, metabolomics primarily provides correlative rather than causal evidence. Although elevated leukotriene levels and pathway enrichment were detected, further functional validation experiments are necessary to establish direct causal relationships. Secondly, key pathways require in vivo experimental verification, such as qPCR and WB assays using lung tissues. Finally, to definitively confirm that Lyn is the direct target of Vas, we plan to conduct the following experiments: (1) Utilize the Lyn kinase activity assay kit to verify the inhibitory effect of Vas on Lyn kinase activity in a cell-free system; (2) Establish MC lines with Lyn gene knockout or kinase inactivation to observe whether Vas loses its inhibitory effect on downstream Syk and MAPK under these conditions. Additionally, to comprehensively evaluate the role of MCs in allergic asthma, in vivo experimental validation will be conducted using methods such as flow cytometry.

## 4. Materials and Methods

### 4.1. Reagents

Vas, extracted and isolated from *A. vasica*, a plant of the genus Adhatoda in the family Acanthaceae, was identified by NMR spectroscopy, with a purity of ≥98%, as determined by HPLC analysis.

Ovalbumin (OVA, 9006-59-1), Dexamethasone acetate (Dex, D4902) were obtained from Sigma-Aldrich. Co., Ltd. (St. Louis, MO, USA). Methacholine (MCH, 62-51-1) was obtained from Spectrum Chemicals (Gardena, CA, USA).

### 4.2. Animals

All animal experiments conducted throughout this study received the necessary approval from the Animal Experimental Ethics Committee affiliated with Yunnan University of Chinese Medicine, with the approval reference number R-062021141 (10 November 2021–9 November 2026). This level of oversight ensures that the experiments adhered to ethical standards surrounding animal research. The subjects used in this experimentation were female BALB/c mice, acquired from SPF (Beijing) Biotechnology Co., Ltd. (Beijing, China), a provider that possesses the requisite animal license number SCXK (Jing) 2019-0010. To maintain the well-being and health of the mice, all subjects were raised in a carefully controlled environment. In this setting, the temperature was consistently maintained at 23 ± 2 °C, while humidity levels were held steady at 55 ± 5%. Such meticulous control of environmental factors is fundamental for promoting the welfare of the animals and obtaining reliable experimental data. Furthermore, the mice were subjected to a 12 h light/dark cycle designed to mimic their natural habitat, which is crucial for their physiological and behavioral health. Before initiating the experiments, a stabilization period of seven days was implemented for the mice. This acclimation phase provided the animals with an opportunity to adjust to their new environment, thereby minimizing stress and anxiety that could otherwise interfere with the results of the study. This careful consideration of the animals’ adaptation process underscores the commitment to ethical standards and the pursuit of high-quality research outcomes.

### 4.3. Experimental Protocols for Allergic Asthma and Vas Intervention

The mouse model of allergic asthma induced by ovalbumin (OVA) was established following the methodology outlined in previous research conducted by Rong et al. in 2024 [4]. In this study, thirty-six BALB/c mice were utilized and randomly allocated into six unique groups for the purpose of investigating the effects of various treatments. The groups included a control that did not receive any intervention, a model which likely presented a specific condition for assessment, and a treatment group that received Dexamethasone at a dosage of 1 mg/kg, known as Dex. Additionally, the groups distinguished by their administration of Vas included one that received a high dose of 100 mg/kg, labeled as Vas-H, another that was given a medium dose of 50 mg/kg, referred to as Vas-M, and a third group that was administered a lower dosage of 25 mg/kg, identified as Vas-L. Each group was structured to allow for a comprehensive evaluation of the effects associated with the treatments in contrast to the control and model. The model and drug treatment groups received intraperitoneal injections of 0.2 mL of a sensitization solution, which contained 0.5 mg/mL OVA and 2.5 mg/mL aluminum hydroxide, on days 1, 8, and 15. Following the sensitization phase, the mice underwent aerosol inhalation of a 1% OVA solution for 30 min per session on days 21 and 27. In contrast, the control was treated with physiological saline. Thirty minutes prior to aerosol exposure, mice in the Dex and Vas groups were administered their respective treatments via gavage, while the control and model groups received equivalent volumes of physiological saline using the same method.

### 4.4. AHR Test

24 h following the completion of the final OVA challenge, a total of 36 mice were evaluated for AHR, with six mice per group. The assessment involved measuring the average baseline readings over a duration of three minutes, utilizing the Flexi Vent system from WBP, EMMS, based in England (Alton, UK). For the purpose of inducing airway contraction, atomization was administered using escalating doses of MCH, specifically at concentrations of 0, 6.25, 12.5, 25, 50, and 100 mg/mL, all dissolved in sterile saline. The responses to these doses were also recorded over a three-minute period. To quantify the level of airway reactivity among the subjects, the Penh was calculated. This index served as a metric to represent the degree of airway contraction, thereby reflecting the intensity of hyperreactivity experienced by the mice [38,39].

### 4.5. Measurement of OVA-sIgE and t-IgE Levels in Serum

A syringe was employed to collect blood samples from the abdominal aorta following the final excitation. The collected blood samples underwent a centrifugation process at a temperature of 4 °C, where they were spun at 1500× *g* for a duration of 10 min. Subsequently, the levels of OVA-sIgE and t-IgE (MM-48374M1, MM-0056 M1) (Jiangsu Meimian industrial Co., Ltd., Yancheng, China) were quantified using ELISA techniques. The absorbance readings for these assays were recorded at a specific wavelength of 450 nanometers, utilizing a microplate reader for precise measurements.

### 4.6. BALF Preparation and Measurement of Cytokine Levels in BALF

In the experimental procedure, the left lung of the mouse was selectively occluded to prevent airflow, while the right lung was subjected to a gentle lavage process. This lavage was performed twice through a tracheal tube and involved the use of a total volume of 0.5 mL of phosphate-buffered saline (PBS) to ensure effective washing of the lung tissue. Following the lavage, the BALF was subjected to centrifugation at a force of 1200× *g* for a duration of 10 min. After centrifugation, the supernatant was carefully collected for subsequent analysis, which was conducted using the ELISA technique to assess the components present in the lavage fluid.

IL-4 (MM-0165 M1), IL-5 (MM-0164 M1), IL-9 (MM-0162 M1), IL-13 (MM-0173 M1), IL-25 (MM-1063 M1), IL-33 (MM-0935 M1), IL-36 (MM-0925 M1), TNF-α (MM-0132M2), TSLP (MM-444481 M1) and MUC5AC (JM-02501M1) (Jiangsu Meimian industrial Co., Ltd., Yancheng, China) levels in BALF were quantified by ELISA.

### 4.7. Histopathological Evaluation of Lungs

The lungs of the mice were excised and preserved in a 10% buffered formalin solution. Following the fixation process, 3 µM thick sections of paraffin were prepared and subjected to H&E staining in order to assess the infiltration of inflammatory cells and the thickening of alveolar walls. To detect the presence of mast cell infiltration in the lung tissue, TB staining was employed. Furthermore, PAS staining was performed on the lung samples to examine goblet cell hyperplasia and the excessive production of mucus. The tissues were examined using optical microscopy, and blinded pathological scoring was conducted employing PAS and H&E staining methods, as outlined by Dong et al. [49]. Furthermore, TB staining scoring was performed in accordance with the methodology described by He et al. [50].

### 4.8. Lung Immunohistochemistry

The expressions of IL-4 (BS-0581R), IL-5 (BS-1318R), IL-9 (BS-2428R), IL-13 (BS-0560R), IL-25(BS-10943R), IL-33(BS-2208R), TNF-α (BS-10802R), and TSLP (BS-2208R) (Beijing Biosynthesis Biotechnology Co., Ltd., Beijing, China) in lung tissues were measured via immunohistochemical (IHC) analysis. Microscopic examination was conducted to identify histopathological alterations, and images were captured for subsequent analysis. The results were assessed using ImageJ software (version 1.8.0; National Institutes of Health, Starkville, MS, USA).

### 4.9. LC-MS Non-Metabolomics Detection of the Lung Tissue of Asthmatic Mice

The control, model, and Vas-H each randomly selected six mice lung tissues for metabolite extraction and non-targeted metabolic group analysis, which was conducted by Shanghai Majorbio Bio-pharm Technology Co., Ltd. (Shanghai, China). Principal component analysis of the metabolic levels of the samples was performed using the R package ROPLS (Version 1.6.2). The differences between the mutant degrees and the sample groups were assessed [51]. The R package ROPLS (Version 1.6.2) was utilized for the statistical analysis of the metabolic group dataset, with an emphasis on identifying metabolites that exhibit differential expression. Detailed experimental methods and data processing procedures are provided in Supplementary Material S2.

### 4.10. Virtual Molecular Docking

The AlphaFold3 (SyK, ERK1, ERK2, JNK) or RCSB PDB database (Lyn: https://www.rcsb.org/structure/2ZV9 (accessed on 16 December 2023), PDB ID: 2ZV9; P38: https://www.rcsb.org/structure/6SP9, PDB ID: 6SP9) was used to obtain the crystal structures corresponding to the six proteins, which were then processed using the Protein Preparation Wizard module of the Schrodinger Maestro 14.3 software to perform protein preprocess, regenerate the states of native Vas, optimize H-bond assignment, minimize protein energy, and remove water. The two-dimensional SDF structure file of the compound Vas was meticulously processed using the LigPrep module available in the Schrödinger software suite. This processing resulted in the generation of all possible three-dimensional chiral conformations of the compound, allowing for a comprehensive exploration of its structural variations. Following this, the SiteMap module within Schrödinger was employed to accurately identify the optimal binding site for the compound. This step is crucial, as it determines the specific location where the ligand is most likely to interact with its target protein. In conjunction with this, the Receptor Grid Generation module was utilized to create an appropriately sized enclosing box that adequately encompasses the identified binding site, ensuring that the docking simulations are both precise and relevant. The docking studies for the ligand compound Vas were then conducted at the active sites of six different proteins, using the high-precision XP docking method to yield the most accurate results. In this context, a lower docking score is indicative of reduced binding free energy between the compound and the target proteins. This observation implies that a lower score correlates with an increased stability of the binding interaction, demonstrating the potential effectiveness of the compound in stabilizing its interaction with the protein targets. Furthermore, the analysis of the binding dynamics of the ligand compound Vas at the active sites of these six proteins was conducted through the MM-GBSA approach [52]. The binding free energies, quantified as MM-GBSA dG Bind, reinforce this relationship, indicating that a decrease in binding free energy corresponds to improved binding stability. This analysis is essential for elucidating the strength of the interactions and the overall efficacy of the ligand in its biological context.

### 4.11. Cell Culture

RBL-2H3 cells have been widely utilized in research focused on the interaction between IgE and FcεRI receptors. RBL-2H3 (GDC0213, American Type Culture Collection, Manassas, VA, USA) cells were grown in Eagle’s Minimum Essential Medium (EMEM), which was enriched with 100 U/mL of penicillin and 100 µg/mL of streptomycin to prevent bacterial contamination, as well as 15% fetal bovine serum (FBS) to provide essential nutrients for optimal cell growth. The cultures were consistently maintained in a controlled environment, set at a temperature of 37 °C with a carbon dioxide level of 5%, using a CO_2_ incubator to ensure ideal growth conditions for the cells.

### 4.12. Measurement of β-Hexosaminidase and Histamine in RBL-2H3 Cells

The degranulation process of RBL-2H3 cells was evaluated by quantifying the activity of the enzyme β-HEX and histamine, which is stored in granules and later released into the cell supernatants. Keto prevents MC degranulation by inhibiting intracellular calcium ion mobilization and other pathways, significantly reducing the release of mediators such as histamine. Consequently, keto was chosen as the positive control group [53]. RBL-2H3 cells were incubated overnight in a 96-well plate at 37 °C with 20 µL of DNP-IgE at a concentration of 1 µg/mL, using a density of 1.5 × 10^4^ cells/mL. The following day, the cells received treatments either with various concentrations of high-dose Vas (100 μM, Vas-H), medium-dose Vas (50 μM, Vas-M), low-dose Vas (25 μM, Vas-L), or keto fumarate (60 μM, Keto) (Sigma-Aldrich, St. Louis, MO, USA) for a duration of 1 h. After treatment, the cells underwent three washes with PIPES buffer (Beyotime, Beyotime Biotechnology, shanghai, China). The stimulation phase included the addition of 100 μL of DNP-HSA (40 ng/mL) in PIPES, followed by incubation for 1 h. Subsequently, 50 μL of the cell supernatant was placed into a fresh 96-well plate, combined with 50 μL of the substrate solution (4-nitro-phenyl N-acetyl-β-d-glucosaminide), and incubated for another hour at 37 °C. To terminate the reaction, 200 μL of a Na_2_CO_3_–NaHCO_3_ buffer was added, and the resultant absorbance was recorded at 570 nm using a microplate reader, facilitating the examination of the inhibition of release induced by Vas. The RBL-2H3 cell supernatant was then analyzed for histamine release using the histamine (H171) (Nanjing Jiancheng Bioengineering Institute, Nanjing, China) ELISA kit.

### 4.13. Measurement of IL-4, IL-5, IL-9, IL-13, and IL-33 from RBL-2H3 Cells

Cells of the RBL-2H3 line, at a density of 1.5 × 10^4^ cells/mL, were seeded in a 96-well plate. With the exception of the control group, all other groups received sensitization treatment with 20 µL of DNP-IgE at a concentration of 1 µg/mL overnight. On the following day, RBL-2H3 cells were pretreated with Keto and Vas, whereas the control and model groups received equivalent amounts of complete medium. After an hour, the cells were rinsed three times using PIPES buffer. Each well was subsequently stimulated by the addition of 100 µL of DNP-HSA (40 ng/mL), with the normal group being replaced by PIPES (+) buffer. Thirty minutes later, the supernatant from the RBL-2H3 cells was collected and transferred to a 96-well plate, where the levels of IL-4 (MM-0191R1), IL-5 (MM-0094R1), IL-9 (MM-0172R1), IL-13 (MM-0085R1), and IL-33 (MM-6146R1) (Jiangsu Meimian Industrial Co., Ltd., Xuzhou, China) were measured according to the specifications provided by the respective ELISA kits.

### 4.14. Measurement and Observation of Intracellular Ca^2+^ Concentrations

Calcium concentrations (Ca^2+^) were assessed utilizing the calcium-sensitive fluorescence indicator Fluo-3AM, following established protocols [54]. RBL-2H3 cells were prepared at a density of 2 × 10^5^ cells/mL and sensitized overnight with DNP-IgE at a concentration of 1 μg/mL in a 12-well plate. Subsequently, the cells underwent pretreatment with varying concentrations of Vas or keto for one hour. After washing the cells three times with D-Hank’s Balanced Salt Solution (HBSS) (Biosharp, Biosharp Biological Technology, Beijing, China), they were incubated in a Fluo-3AM solution containing 0.05% Pluronic F127 in HBSS at 37 °C in the dark for 40 min. Following the removal of unbound Fluo-3AM, the cells were treated with anti-DNP-HSA at a concentration of 40 µg/mL. Fluorescence measurements were conducted by utilizing an excitation wavelength set at 488 nanometers, while the corresponding emission wavelength was established at 525 nanometers. To capture the resulting images, a fluorescence microscope (Leica DM18, produced by Leica Microsystems in Wetzlar, Germany) was employed, allowing for direct visualization of the fluorescence emitted from the sample.

### 4.15. Western Blot Analysis of RBL-2H3 Cells

RBL-2H3 cells, at a density of 4.5 × 10^5^ cells/mL, were seeded in a 6-well plate and exposed to 400 μL of 1 μg/mL DNP-IgE, followed by incubation overnight at 37 °C. After conducting three washes with PIPES buffer, varying concentrations of Vas or keto were administered to the cells for an hour. The cells were then stimulated with DNP-HSA at a concentration of 40 ng/mL for an additional hour. Following stimulation, the cells underwent three washes with PBS, after which RIPA lysis buffer was added, and the samples were kept on ice for 30 min. Subsequently, the lysates were centrifuged at 10,000× *g* for 15 min at 4 °C. The protein concentrations in the whole cell lysates were quantified using a BCA protein assay. Equal volumes of each sample were subjected to SDS-polyacrylamide gel electrophoresis (SDS-PAGE), after which the separated proteins were transferred onto polyvinylidene fluoride membranes (PVDF). The membrane was blocked for one hour at room temperature with TBST (Tris Buffered Saline with Tween 20) containing 5% skim milk, followed by an overnight incubation at 4 °C with primary antibodies such as p-Lyn (ab278639), Lyn (ab32398), p-Syk (ab300398), Syk (ab40781), p-ERK1/2 (ab76299), ERK1/2 (11257-1-AP), p-JNK (80024-1-RR), JNK (24164-1-AP), p-P38 (28796-1-AP), P38 (14064-1-AP), and GAPDH (10494-1-AP) sourced from Abcam (Cambridge, UK) and Proteintech (Chicago, IL, USA). After thoroughly washing the membrane with TBST, secondary antibodies—namely horseradish peroxidase-conjugated goat anti-rabbit and anti-mouse IgG-were applied for two hours. Band visualization was performed using a K13059 imaging system from Analytical Instruments in Jena, Germany. The band intensities of Western blot were quantified using ImageJ software and normalized to GAPDH as an internal control.

### 4.16. Statistical Analysis

The data are presented as means and standard errors of the mean (SEM) to provide a comprehensive understanding of the results. All statistical analyses were conducted using GraphPad Prism version 8.0.2 software, developed by GraphPad Software, Inc. (San Diego, CA, USA). To analyze the grayscale values obtained from Western blotting, ImageJ software was utilized to ensure accurate measurement and interpretation of the data. The Shapiro–Wilk test was employed to assess the normality of the data. For data that were normally distributed, one-way analysis of variance (ANOVA) was performed to evaluate the treatment effects, followed by Bonferroni’s correction to adjust for multiple comparisons. A *p*-value of less than 0.05 was established as the threshold for determining statistical significance, indicating that the research findings exhibit meaningful differences [55,56,57].

## 5. Conclusions

**Figure 8:**
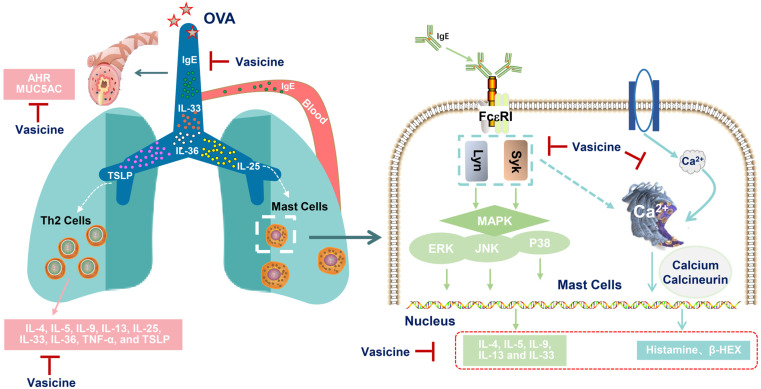
Mechanism diagram of Vas intervention in allergic asthma.

In summary, Vas may alleviate inflammatory responses and airway hyperresponsiveness in allergic asthmatic mice while reducing airway mucus MUC5AC secretion. Integrated in vivo and in vitro data suggest that the anti-asthmatic effect of Vas may partially stem from its inhibition of the mast cell FcεRI/Lyn/Syk/MAPK signaling pathway, thereby reducing mast cell degranulation and Th2 inflammatory cytokine release, and intervening in the pathological progression of asthma (Figure 8). These findings lay the foundation for future research on screening potential anti-asthmatic compounds from natural products.

## Figures and Tables

**Table 1:** XP and MM-GBSA results.

Compound	Target	XP Gscore	MM-GBSA dG Bind (kcal/mol)
Vas	Lyn	−5.012	−35.49
PP2		−8.468	−50.45
Vas	Syk	−4.463	−29.84
Sovleplenib		−3.198	−38.40
Vas	ERK1	−4.043	−32.43
BVD-523		−4.944	−18.89
Vas	ERK2	−4.400	−29.41
BVD-523		−3.751	−31.40
Vas	JNK	−3.080	−10.92
SP600125		−2.660	−9.956
Vas	P38	−4.308	−32.81
SB4		−5.469	−28.56

## Data Availability

The data that has been analyzed and discussed in this study is comprehensively included within the main body of the article as well as in the Supplementary Materials. Should you have any additional questions or require further clarification regarding the data, you are encouraged to reach out directly to the corresponding authors, who will be pleased to assist you.

## Supplementary Materials

The following supporting information can be downloaded at: https://www.mdpi.com/article/10.3390/ph19010190/s1, Supplementary Material S1: AUC of the dose–-response curve; Supplementary Material S2: LC-MS non-metabolomics detection of the lung tissue of asthmatic mice; Supplementary Material S3: The intersecting metabolic pathways between the control group and the model group; Supplementary Material S4: The intersecting metabolic pathways between the model group and the Vas group; Supplementary Material S5: The specific binding mode diagrams between each target protein and its corresponding positive control compound; Supplementary Material S6: Western blot results.

## Abbreviations

The following abbreviations are used in this manuscript:

Vas	Vasicine
OVA	Ovalbumin
Dex	Dexamethasone
ELISA	enzyme-linked immunosorbent assay
TSLP	thymic stromal lymphoprotein
TNF-α	tumor necrosis factor
KEGG	Kyoto Encyclopedia of Genes and Genomes
UPLC-Q-TOF-MS	ultra performance liquid chromatography-quadrupole-time of flight-mass spectrometry
PLS-DA	partial least squares discriminant analysis
OPLS-DA	orthogonal partial least squares-discriminant analysis
MAPK	mitogen-activated protein kinase
XP	eXtended Precision
Keto	Ketotifen Fumarate
AHR	Abbreviations
BALF	Bronchoalveolar lavage fluid
