# Supplementary material for: Vasicine Attenuates Allergic Asthma by Suppressing Mast Cell Degranulation and Th2 Inflammation via Modulation of the FcεRI/Lyn + Syk/MAPK Pathway
Source: Pharmaceuticals (Basel). 2026 Jan 22;19(1):190. doi: 10.3390/ph19010190 (PMC12845140; doi:10.3390/ph19010190)
Supplement: Supplementary file 1 [file pharmaceuticals-19-00190-s001.zip › Supplementary Material S6-Western blot results/Supplementary Material S6-Western blot results-band intensity ratios.pdf]

p-Lyn/Lyn band intensity ratios (n=3)

Control	Model	Keto	Vas-25	Vas-50	Vas-100
0.619095188	1.305855222	0.879037084	0.755497121	0.781308706	0.557103668
0.652266981	1.338606044	0.606919825	0.983187439	0.612674314	0.671643568
0.792344322	1.22226275	0.808139755	0.752660928	0.847593515	0.67553758

p-Syk/Syk band intensity ratios (n=3)

Control	Model	Keto	Vas-25	Vas-50	Vas-100
0.629038	1.444217	0.71399	0.796203	0.506427	0.440111
0.586168	1.242412	0.596653	0.620813	0.338021	0.309202
0.434035	1.4119	0.625603	0.687847	0.406814	0.344239

p-ERK/ERK band intensity ratios (n=3)

Control	Model	Keto	Vas-25	Vas-50	Vas-100
0.250266389	1.16007797	0.85284262	0.783083492	0.863149966	0.582584954
0.13688651	1.224867168	0.812608971	0.852412044	0.90185033	0.858955517
0.39840404	1.291325858	0.89208169	0.965612654	0.880046454	0.779459832

p-JNK/JNK band intensity ratios (n=3)

Control	Model	Keto	Vas-25	Vas-50	Vas-100
0.540702431	1.067328482	0.744629009	0.769761241	0.727750789	0.751762948
0.571949945	1.084442776	0.76653095	0.667501933	0.634803012	0.727762423
0.525918146	1.017270652	0.822408442	0.808249276	0.723691795	0.666277328

p-P38/P38 band intensity ratios (n=3)

Control	Model	Keto	Vas-25	Vas-50	Vas-100
0.305446943	1.1352418	0.91199519	0.745628493	0.251319851	0.24551901
0.220121051	1.066793315	0.60330701	0.832315111	0.363292052	0.379000824
0.490651431	1.156929822	0.563515785	0.823721684	0.432134246	0.359794588
