# Supplementary material for: Vasicine Attenuates Allergic Asthma by Suppressing Mast Cell Degranulation and Th2 Inflammation via Modulation of the FcεRI/Lyn + Syk/MAPK Pathway
Source: Pharmaceuticals (Basel). 2026 Jan 22;19(1):190. doi: 10.3390/ph19010190 (PMC12845140; doi:10.3390/ph19010190)
Supplement: Supplementary file 1 [file pharmaceuticals-19-00190-s001.zip › Supplementary Material S5-The specific binding mode diagrams between each target protein and its corresponding positive control compound.pdf]

The specific binding mode diagrams between each target protein and its corresponding positive control compound

Nevertheless, we have supplemented our analysis using the same method. Specifically, we conducted molecular docking (XP docking) and binding free energy (MM-GBSA) analysis for common inhibitors (positive control compounds) associated with each target protein. The supplementary data and comparative results regarding vasicine's binding properties are compiled below to provide more comprehensive evidence of vasicine's binding capacity and stability with each target protein. To enhance the binding characteristic data of positive control compounds with target proteins (Table 1), the figure 1 presents 2D and 3D diagrams of docking interactions between positive control compounds and Lyn (A), Syk(B), ERK1 (C), ERK2 (D), JNK (E), and P38 (F) proteins. We selected classic and commonly used inhibitors for each target as positive controls. Using the same experimental methods as for vasicine (XP docking + MM-GBSA analysis), we examined their binding characteristics with the corresponding target proteins. The specific results are displayed in the table below.

Table 1. XP and MM-GBSA results.

Compound	Target	XP Gscore	MM-GBSA dG Bind (kcal/mol)
Vas	Lyn	-5.012	-35.49
PP2		-8.468	-50.45
Vas	Syk	-4.463	-29.84
Sovleplenib		-3.198	-38.40
Vas	ERK1	-4.043	-32.43
BVD-523		-4.944	-18.89
Vas	ERK2	-4.400	-29.41
BVD-523		-3.751	-31.40
Vas	JNK	-3.080	-10.92
SP600125		-2.660	-9.956
Vas	P38	-4.308	-32.81
SB4		-5.469	-28.56

A

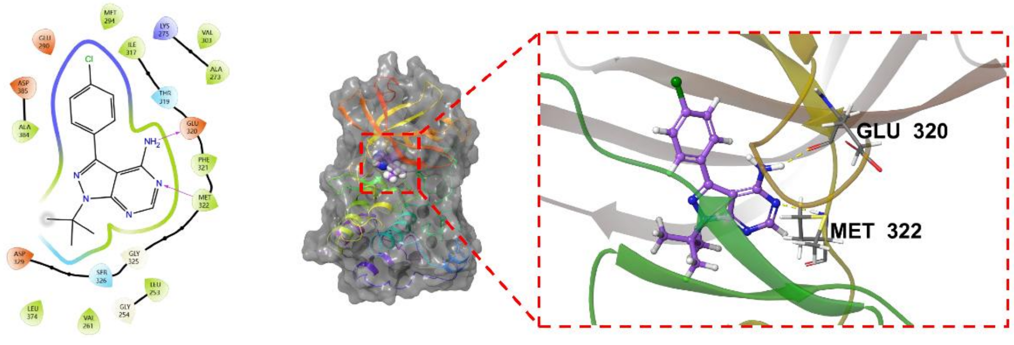

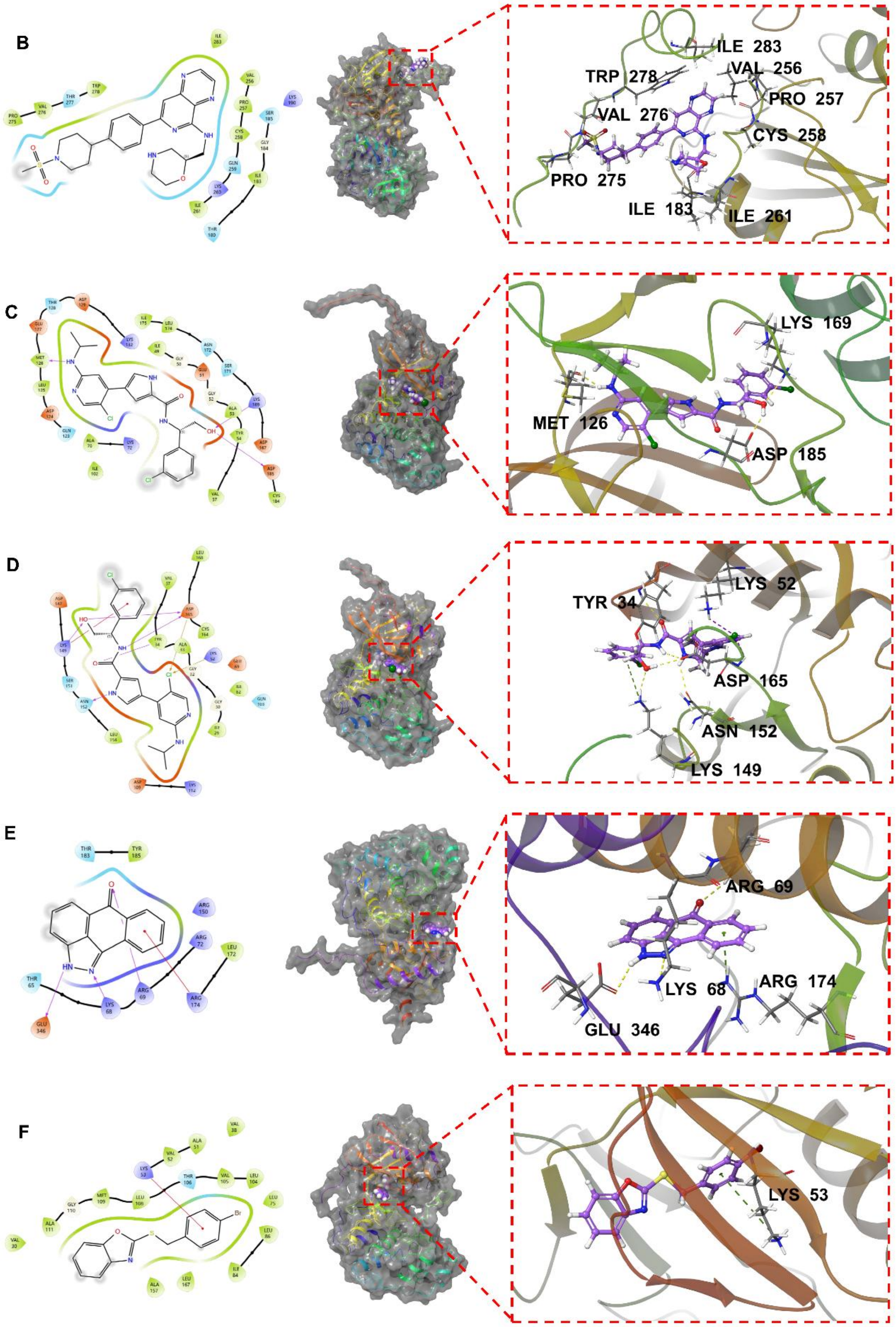

Figure 1 2D and 3D diagrams of docking interactions between positive control compounds and Lyn (A), Syk(B); ERK1 (C), ERK2 (D), JNK (E), and P38 (F) proteins. Note: Yellow represents hydrogen bonds, lavender represents salt bridges, and green represents π cation bonds.

Based on the supplementary data and the original data, we conducted a targeted

comparison of the binding characteristics between vasicine and the corresponding positive control compounds for the target proteins.

1.For the Lyn target, the positive control compound PP2 exhibited significantly lower XP docking scores (-8.486) and binding free energy (-50.45 kcal/mol) compared to vasicine (docking score -5.012, binding free energy -35.49 kcal/mol), indicating that PP2 has superior binding stability with Lyn than vasicine. However, vasicine still demonstrated relatively stable binding capability despite its lower docking score and binding free energy, which validates its potential binding activity to the Lyn target. From the perspective of binding patterns, both compounds can deeply penetrate the active pocket of the Lyn protein: vasicine forms hydrophobic interactions with Lyn protein residues VAL261, LEU374, PHE321, MET322, etc., and establishes one hydrogen bond each with residues ASP329 and SER326; PP2 establishes hydrophobic interactions with Lyn protein residues MET294, ILE317, PHE321, MET322, LEU253, etc., and forms one hydrogen bond each with residues GLU320 and MET322. Both compounds achieve stable binding through the synergistic effects of hydrophobic interactions and hydrogen bonds, with PP2 involving more residues in hydrophobic interactions, which may be a reason for its superior binding stability.

2.The positive control compound Sovleplenib achieved an XP docking score of -3.198 with SYK and an MM-GBSA binding free energy of -38.40 kcal/mol. The relatively low docking score and binding free energy indicate good binding stability with SYK. Vasicine showed XP docking scores and MM-GBSA binding free energies of -4.463 and -29.84 kcal/mol with SYK, respectively. Both values are at relatively low levels, demonstrating that Vasicine can also stably bind with SYK. The binding mode analysis revealed that the positive control compound SP600125 could be embedded in the active pocket of JNK protein, forming hydrophobic interactions with residues TYR185 and LEU172. Simultaneously, it formed one hydrogen bond each with GLU346, LYS68, and ARG69, as well as one π-cation bond with ARG174. These multiple interactions collectively maintained its binding conformation with JNK. After embedding into the SYK active pocket, Vasicine can form hydrophobic interactions with residues such as ILE283, LEU122, PRO166, PRO257, and CYS258, while forming two hydrogen bonds with LYS260. The stable binding with SYK is achieved through the synergistic effects of hydrophobic interactions and hydrogen bonds.

3.The positive control compound BVD-523 exhibits relatively unstable binding with ERK1, as evidenced by a docking score of -4.944 and a binding free energy of -18.89 kcal/mol. In contrast, vasicine demonstrates a lower docking score of -4.043 and a significantly reduced binding free energy of -32.43 kcal/mol with ERK1. This indicates that vasicine possesses superior binding stability with ERK1 compared to the positive control compound BVD-523, thereby highlighting vasicine's favorable binding activity towards the ERK1 target. The binding mode analysis reveals that both compounds can deeply penetrate the active pocket of the ERK1 protein. Peganine forms a hydrogen bond with the residue ASN172 of the ERK1 protein and establishes a salt bridge with the residue ASP129. Conversely, BVD-523 forms hydrophobic interactions with the residues ILE102, ALA70, LEU125, MET126, and ILE49 of the ERK1 protein, in addition to forming one hydrogen bond each with the residues ASP185, LYS169, and MET126. Although BVD-523 exhibits

hydrophobic interactions and multiple hydrogen bonds, it lacks strong interactions such as salt bridges. In contrast, the synergistic effect of hydrogen bonding and salt bridge formation in peganine significantly enhances binding stability, making it superior to BVD-523.

4.The docking score of the positive control compound BVD-523 (-3.751) was slightly higher than that of vasicine (-4.400), while its binding free energy (-31.40 kcal/mol) was marginally lower than that of vasicine (-29.41 kcal/mol). Both compounds exhibited relatively low docking scores and binding free energies, indicating that the binding stability of vasicine with ERK2 is comparable to that of the positive control compound BVD-523. This finding further corroborates the binding activity of vasicine to the ERK2 target. The binding mode analysis revealed that both compounds can deeply penetrate the active pocket of the ERK2 protein. Vasicine forms hydrophobic interactions with ERK2 residues ILE29, ALA50, LEU105, and MET106, while also forming one hydrogen bond with residue MET106 and one hydrogen bond plus a salt bridge with residue ASP109. In contrast, BVD-523 establishes hydrophobic interactions with ERK2 residues VAL37, CYS164, TYR34, ALA33, and ILE29, exhibiting more extensive binding interactions. These include forming one hydrogen bond each with residues TYR34 and ASN152, one hydrogen bond plus a π-cation interaction with residue LYS149, two hydrogen bonds and one halogen bond with residue ASP165, and one halogen bond with residue LYS52. Although BVD-523 exhibits a greater diversity of interaction types, vasicine achieves comparable binding stability to BVD-523 through the synergistic effects of hydrophobic interactions, hydrogen bonds, and salt bridges.

5.The positive control compound SP600125 exhibits unstable binding with JNK, as evidenced by a docking score of -2.660 and a binding free energy of -9.56 kcal/mol. In comparison, vasicine displays a slightly lower docking score of -3.080; however, it maintains a relatively high binding free energy of -10.92 kcal/mol with JNK. Both compounds demonstrate unstable binding, consistently supporting the conclusion that JNK is not the preferential binding target for vasicine. Both compounds interact significantly with the active pocket of the JNK protein: vasicine establishes a hydrogen bond and a salt bridge with the residue GLU346, alongside π-cation interactions with residues LYS68 and PHE180. In contrast, SP600125 forms hydrophobic interactions with residues TYR185 and LEU172, while simultaneously establishing hydrogen bonds with residues GLU346, LYS68, and ARG69, and a π-cation interaction with residue ARG174. Despite the formation of multiple interactions, the consistently high binding free energies suggest weak synergistic effects of these interactions, which fail to achieve stable binding. This further corroborates the non-dominant nature of the JNK target.

6.The docking score of the positive control compound SB4 (-5.469) was lower than that of vasicine (-4.308), while its binding free energy (-28.56 kcal/mol) was slightly higher than that of vasicine (-32.81 kcal/mol). Both compounds exhibited relatively low docking scores and binding free energies, indicating similar binding stability. Notably, vasicine demonstrated a lower binding free energy, suggesting its superior binding capability to the p38 target, which further reinforces the relevant conclusions. In terms of binding mode, both compounds penetrate deeply into the active pocket of the p38 protein. Vasicine forms hydrophobic interactions with p38 protein residues LEU167, ALA157, VAL30, among

others, while also establishing a hydrogen bond with residue SER32 and a salt bridge with residue ASP168. Conversely, SB4 establishes hydrophobic interactions with p38 protein residues VAL105, LEU104, LEU75, LEU86, ILE84, and others, and forms a π-cation bond with residue LYS53. Vasicine compensates for its relatively fewer hydrophobic interaction residues through the additional contributions of hydrogen bonding and salt bridge formation, ultimately achieving superior binding free energy.

The results of molecular docking and binding mode analysis jointly confirm that Vas can target Lyn, Syk, p38, and ERK family kinases, demonstrating distinct target specificity in binding activity. It exhibits superior binding stability with ERK1 and p38 compared to the positive control inhibitors, shows comparable binding activity to Lyn and ERK2 as the positive control, and displays no significant binding capacity to JNK. From a molecular mechanisms perspective, Vas stably embeds into the active pockets of target proteins, forming hydrophobic interactions with specific amino acid residues while synergizing with polar interactions such as hydrogen bonds and salt bridges to establish stable protein-ligand complexes. The binding residues and interaction types vary across different targets, with this structural specificity being the core determinant of differential binding stability among targets. These findings not only elucidate the binding characteristics of Vas with immune-inflammatory-related kinase targets at the molecular level but also identify its predominant target sites. This provides crucial theoretical foundations and target directions for further investigation into Vas's regulation of kinase signaling pathways and its biological functions in intervening allergic asthma.
