# Supplementary material for: Vasicine Attenuates Allergic Asthma by Suppressing Mast Cell Degranulation and Th2 Inflammation via Modulation of the FcεRI/Lyn + Syk/MAPK Pathway
Source: Pharmaceuticals (Basel). 2026 Jan 22;19(1):190. doi: 10.3390/ph19010190 (PMC12845140; doi:10.3390/ph19010190)
Supplement: Supplementary file 1 [file pharmaceuticals-19-00190-s001.zip › Supplementary Material S2-LC-MS non-metabolomics detection of the lung tissue of asthmatic mice.pdf]

LC-MS non-metabolomics detection of the lung tissue of asthmatic mice

1. Metabolite Extraction

Solid sample:

50 mg solid sample was added to a 2 mL centrifuge tube and a 6 mm diameter grinding bead was added. 400 μL of extraction solution (methanol: water = 4:1 (v:v)) containing 0.02 mg/mL of internal standard (L-2-chlorophenylalanine) was used for metabolite extraction. Samples were ground by the Wonbio-96c (Shanghai wanbo biotechnology co., LTD) frozen tissue grinder for 6 min (-10°C, 50 Hz), followed by low-temperature ultrasonic extraction for 30 min (5°C, 40 kHz). The samples were left at -20°C for 30 min, centrifuged for 15 min (4°C, 13000 g), and the supernatant was transferred to the injection vial for LC-MS/MS analysis.

Liquid sample:

100 μL liquid sample was added to a 1.5 mL centrifuge tube with 400 μL solution (acetonitrile: methanol = 1:1(v:v)) containing 0.02 mg/mL internal standard (L-2-chlorophenylalanine) to extract metabolites.The samples were mixed by vortex for 30 s and low-temperature sonicated for 30 min (5°C, 40 KHz)。The samples were placed at -20°C for 30 min to precipitate the proteins.Then the samples were centrifuged for 15 min (4°C, 13000 g).The supernatant was removed and blown dry under nitrogen. The sample was then re-solubilized with 100 μL solution (acetonitrile: water = 1:1) and extracted by low-temperature ultrasonication for 5 min (5°C, 40 KHz), followed by centrifugation at 13000 g and 4°C for 10 min.The supernatant was transferred to sample vials for LC-MS/MS analysis.

2. Quality control sample

As a part of the system conditioning and quality control process, a pooled quality control sample (QC) was prepared by mixing equal volumes of all samples. The QC samples were disposed and tested in the same manner as the analytic samples. It helped to represent the whole sample set, which would be injected at regular intervals (every 5-15 samples) in order to monitor the stability of the analysis.

3. (UHPLC-MS/MS) analysis

The LC-MS/MS analysis of sample was conducted on a Thermo UHPLC-Q Exactive HF-X system equipped with an ACQUITY HSS T3 column (100 mm × 2.1 mm i.d., 1.8 μm; Waters, USA) at Majorbio Bio-Pharm Technology Co. Ltd. (Shanghai, China). The mobile phases consisted of 0.1% formic acid in water:acetonitrile (95:5, v/v) (solvent A) and 0.1% formic acid in acetonitrile: isopropanol: water (47.5: 47.5, v/v) (solvent B). The flow rate was 0.40 mL/min and the column temperature was 40℃. The injection volume was 3 μL.

MS conditions: The mass spectrometric data were collected using a Thermo UHPLC-Q Exactive HF-X Mass Spectrometer equipped with an electrospray ionization (ESI) source operating in positive mode and negative mode. The optimal conditions were set as followed: source temperature at 425℃; sheath gas flow rate at 50 arb; Aux gas flow rate at 13 arb; ion-spray voltage floating (ISVF) at -3500V in negative mode and 3500V in positive mode, respectively; Normalized collision energy , 20-40-60 eV rolling for MS/MS. Full MS resolution was 60000, and MS/MS resolution was 7500. Data acquisition was performed with the Data Dependent Acquisition (DDA) mode. The detection was carried out over a mass range of 70-

1050 m/z.

4. Data anlaysis

The UHPLC-MS raw data were converted into the common format by Progenesis QI software (Waters, Milford, USA) through baseline filtering, peak identification, peak integral, retention time correction, and peak alignment. Then, the data matrix containing sample names, m/z, retention time and peak intensities was exported for further analyses. At the same time, the metabolites were identified by searching database, and the main databases were the HMDB (http://www.hmdb.ca/), Metlin (https://metlin.scripps.edu/) and the self-compiled Majorbio Database (MJDB) of Majorbio Biotechnology Co., Ltd. (Shanghai, China). The data matrix obtained by searching database was uploaded to the Majorbio cloud platform (https://cloud.majorbio.com) for data analysis. Fistly, the data matrix was pre-processed, as follows: At least 80% of the metabolic features detected in any set of samples were retained. After filtering, the minimum value in the data matrix was selected to fill the missing value and each metabolic signature was normalized to the sum. To reduce the errors caused by sample preparation and instrument instability, the response intensities of the sample mass spectrometry peaks were normalized using the sum normalization method, to obtain the normalized data matrix. Meanwhile, the variables of QC samples with relative standard deviation (RSD) > 30% were excluded and log10 logarithmicized, to obtain the final data matrix for subsequent analysis. Then, the R package "ropls" (Version 1.6.2) was used to perform principal component analysis (PCA) and orthogonal least partial squares discriminant analysis (OPLS-DA), and 7-cycle interactive validation evaluating the stability of the model. The metabolites with VIP>1, $p<0.05$ were determined as significantly different metabolites based on the Variable importance in the projeciton (VIP) obtained by the OPLS-DA model and the p-value generated by student's t test. Differential metabolites among two groups were mapped into their biochemical pathways through metabolic enrichment and pathway analysis based on KEGG database (http://www. genome.jp/kegg/). These metabolites could be classified according to the pathways they involved or the functions they performed. Enrichment analysis was used to analyze a group of metabolites in a function node whether appears or not. The principle was that the annotation analysis of a single metabolite develops into an annotation analysis of a group of metabolites. Python packages "scipy.stats" (https://docs.scipy.org/doc/scipy/) was used to perform enrichment analysis to obtain the most relevant biological pathways for experimental treatments.

Table 1. Differential metabolites in asthmatic mice model after the treatment of Vas.

NO.	Formula	RT [min]	m/z	Metabolites	Pathway
1	$C_{11}H_{14}NO_6^+$	2.0759	298.1150101	Nicotinic acid ribonucleoside	Nicotinate and nicotinamide metabolism
2	$C_8H_9N_5O_3$	2.277	188.0569452	Succinylaminoimidazole carboxamide	-
3	$C_{15}H_{29}NO_4$	5.4483	288.2173542	L-Octanoylcarnitine	-
4	$C_{10}H_{14}N_2$	5.7485	342.2645122	Anabasine	-

5	$C_{21}H_{38}N_4O_8$	6.2329	516.3070692	Amastatin	-
6	$C_{20}H_{32}O_3$	6.1751	303.2322911	20-Hydroxyeicosatetraenoic acid	Arachidonic acid metabolism; Vascular smooth muscle contraction
7	$C_{10}H_{16}O$	6.0863	305.2476602	Perillyl alcohol	-
8	$C_{17}H_{28}O_3$	5.8559	263.2010391	3-Methyl-5-propyl-2-furannonanoic acid	-
9	$C_{19}H_{37}NO_4$	5.7637	344.2801531	Lauroylcarnitine	-
10	$C_{10}H_{13}N_5O_4$	2.1066	250.0938694	Zidovudine	Bile secretion
11	$C_{14}H_{17}N_5O_8$	2.2693	384.1156555	Succinyladenosine	-
12	$C_{11}H_{12}N_2O_5$	2.2693	252.0731201	5-Hydroxy-*N*-formylkynurenine	Tryptophan metabolism
13	$C_7H_9N_5O$	2.277	162.0776679	7-Aminomethyl-7-carbaguanine	Folate biosynthesis
14	$C_{20}H_{19}NO_7$	1.0503	350.1038549	(4As,5aR,6R,12aR)-1,10,11,12a-tetrahydroxy-6-methyl-3,12-dioxo-4a,5,5a,6-tetrahydro-4H-tetracene-2-carboxamide	-
15	$C_{21}H_{34}O_3$	6.1169	317.2479886	Tetrahydrodeoxycorticosterone	Steroid hormone biosynthesis
16	$C_{23}H_{48}NO_7P$	7.3222	482.3254135	LysoPE	-
17	$C_9H_{16}O_6$	0.599	259.0598961	1,2-O-Isopropylidene-D-glucofuranose	-
18	$C_{10}H_{14}N_5O_7P$	1.8737	348.0709688	Adenosine 2'-phosphate	-
19	$C_4H_6N_4O_3$	0.6648	157.0359317	Allantoin	-
20	$C_{10}H_{14}N_5O_7P$	2.1066	346.0561719	3'-Adenylic Acid	Purine metabolism
21	$C_{13}H_{17}N_5O_6$	2.5491	374.0876048	N(2)-(1-Carboxyethyl)-2'-deoxyguanosine	-
22	$C_5H_5N_5$	2.946	134.0462618	9h-Purin-9-amine	-
23	$C_{20}H_{18}F_3N_3O_3S$	2.946	472.0704823	N-[2-[(R)-[3-Methyl-4-(2,2,2-trifluoroethoxy) pyridin-2-yl]methylsulfinyl]-1H-pyrrol-3-yl]benzamide	-
24	$C_{20}H_{36}O_3$	6.0361	323.2594793	11(R)-HEDE	-
25	$C_{23}H_{36}O_7$	5.8712	445.2213754	Pravastatin	Bile secretion
26	$C_{19}H_{23}N_3O_4S$	5.8314	434.1406063	Hetacillin	-
27	$C_{21}H_{36}O_5$	5.7995	413.2549197	Carboprost	-
28	$C_{13}H_{18}N_4O_3$	5.6914	299.1116664	*N*-alpha-Benzoyl-L-arginine	-
29	$C_6H_{12}O_3$	4.5454	131.0703822	6-Hydroxyhexanoic Acid	-

30	$C_{12}H_{15}N_5O_9S_2$	2.946	482.0260466	Tigemonam	-
31	$C_{13}H_{17}N_5O_6$	2.511	376.0668695	Loxoribine	-
32	$C_5H_{11}O_8P$	2.1066	211.0010624	D-Ribose 1-phosphate	Pentose phosphate pathway; Purine metabolism
33	$C_5H_7NO_3$	1.8697	110.0236609	4-Oxo-L-proline	-
34	$C_{10}H_{16}N_2O_5S$	1.8697	311.0501323	Biotin sulfone	Biotin metabolism
35	$C_9H_{12}N_2O_6$	1.8738	243.062358	Uridine	Pyrimidine metabolism; ABC transporters; Nucleotide metabolism
36	$C_8H_{19}O_2PS_2$	0.6966	287.0530203	Ethoprophos	-
37	$C_{30}H_{47}N_3O_9S$	5.8074	624.2968982	**Leukotriene C4**	**Asthma; FcεRI signaling pathway; Arachidonic acid metabolism**; Bile secretion; Neuroactive ligand-receptor interaction; Serotonergic synapse
38	$C_{25}H_{27}ClN_2O_5$	5.8314	451.143481	3-Carboxylic acid-picumast	-
39	$C_{39}H_{75}O_8P$	7.1392	747.5202224	PA (18:1(11Z)/18:0)	Glycerophospholipid metabolism; Phosphatidylinositol signaling system; Glycerolipid metabolism; Pancreatic cancer; Fat digestion and absorption; cAMP signaling pathway; Phospholipase D signaling pathway; GnRH signaling pathway; Choline metabolism in cancer; FcγR-mediated phagocytosis
40	$C_6H_{12}O_6$	1.0775	217.0118282	D-Galactose	Metabolic pathways; Carbohydrate digestion and absorption; Mineral absorption; Galactose metabolism
