# Supplementary material for: Vasicine Attenuates Allergic Asthma by Suppressing Mast Cell Degranulation and Th2 Inflammation via Modulation of the FcεRI/Lyn + Syk/MAPK Pathway
Source: Pharmaceuticals (Basel). 2026 Jan 22;19(1):190. doi: 10.3390/ph19010190 (PMC12845140; doi:10.3390/ph19010190)
Supplement: Supplementary file 1 [file pharmaceuticals-19-00190-s001.zip › Supplementary Material S1-AUC of the dose-response curve.pdf]

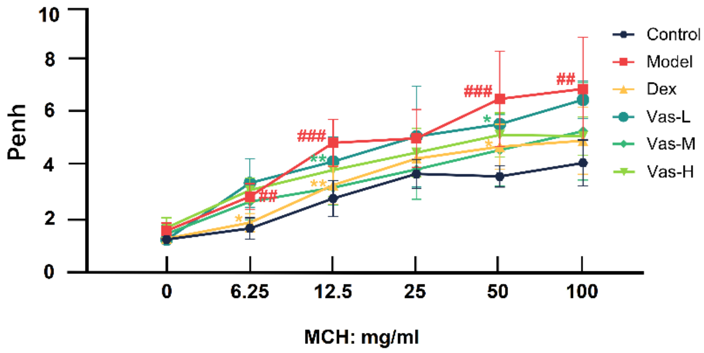

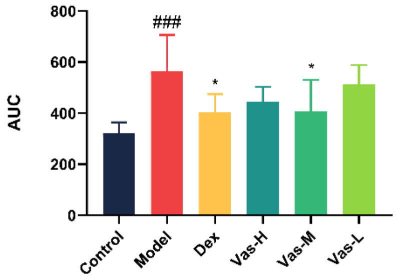

The influence of Vas on AHR in asthmatic mice

AUC of the dose-response curve

Table AUC of the dose-response curve

	Control	Model	Dex	Vas-H	Vas-M	Vas-L
1	265.8	585.5	456.7	452.2	426.4	624.7
2	335.8	671.8	351.9	461	316.5	536.5
3	394.4	499.1	420.8	464.3	489.5	559
4	305.2	778.3	455.2	354.5	603.9	416
5	309.1	411.3	456.4	415.9	286.4	480.9
6	319.6	436.9	288.7	527.6	315.6	458.9
