# Supplementary figures and images for: Vasicine Attenuates Allergic Asthma by Suppressing Mast Cell Degranulation and Th2 Inflammation via Modulation of the FcεRI/Lyn + Syk/MAPK Pathway
Source: Pharmaceuticals (Basel). 2026 Jan 22;19(1):190. doi: 10.3390/ph19010190 (PMC12845140; doi:10.3390/ph19010190)

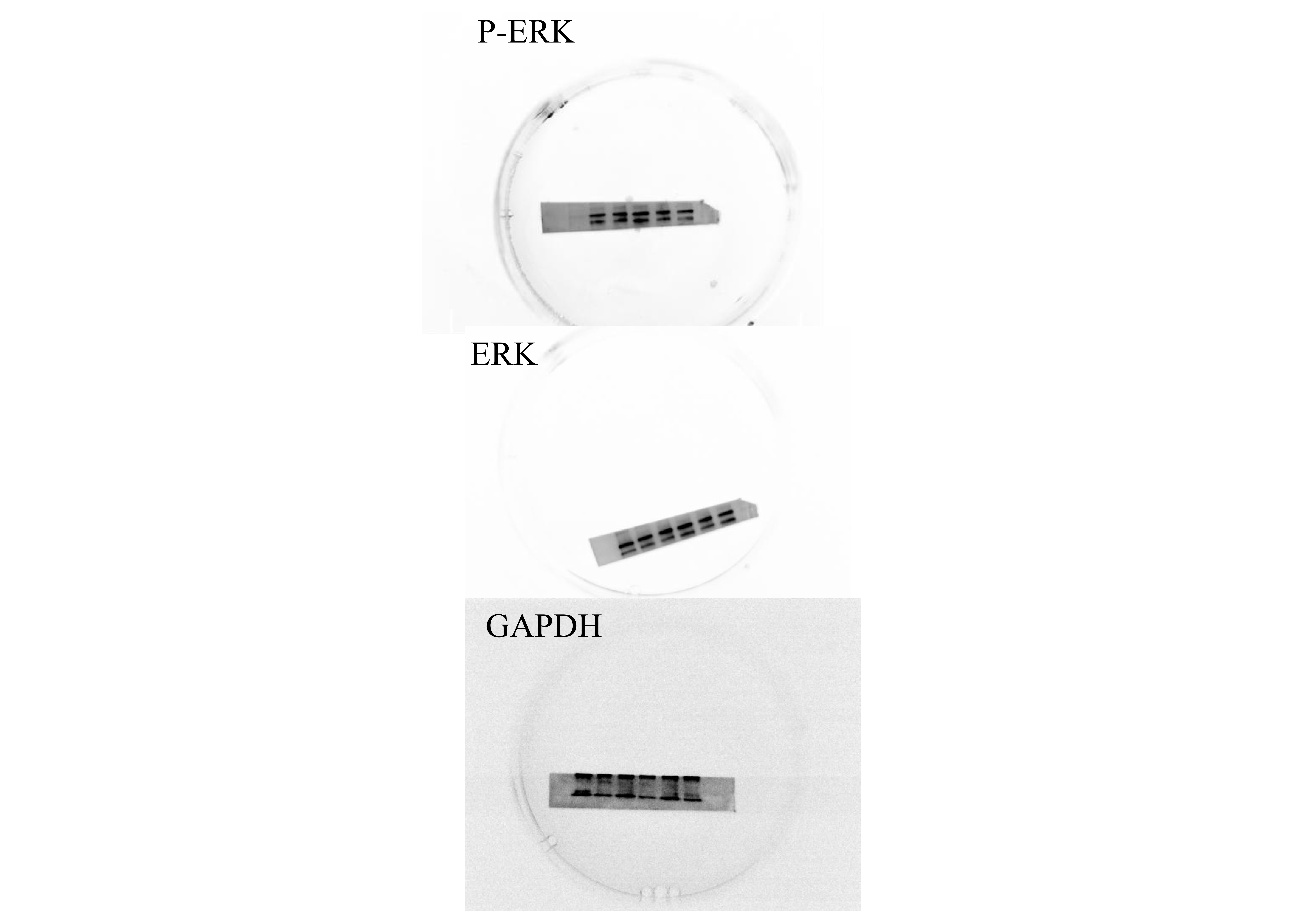

Supplement: Supplementary file 1 [file pharmaceuticals-19-00190-s001.zip › Supplementary Material S6-Western blot results/ERK-1.tif]

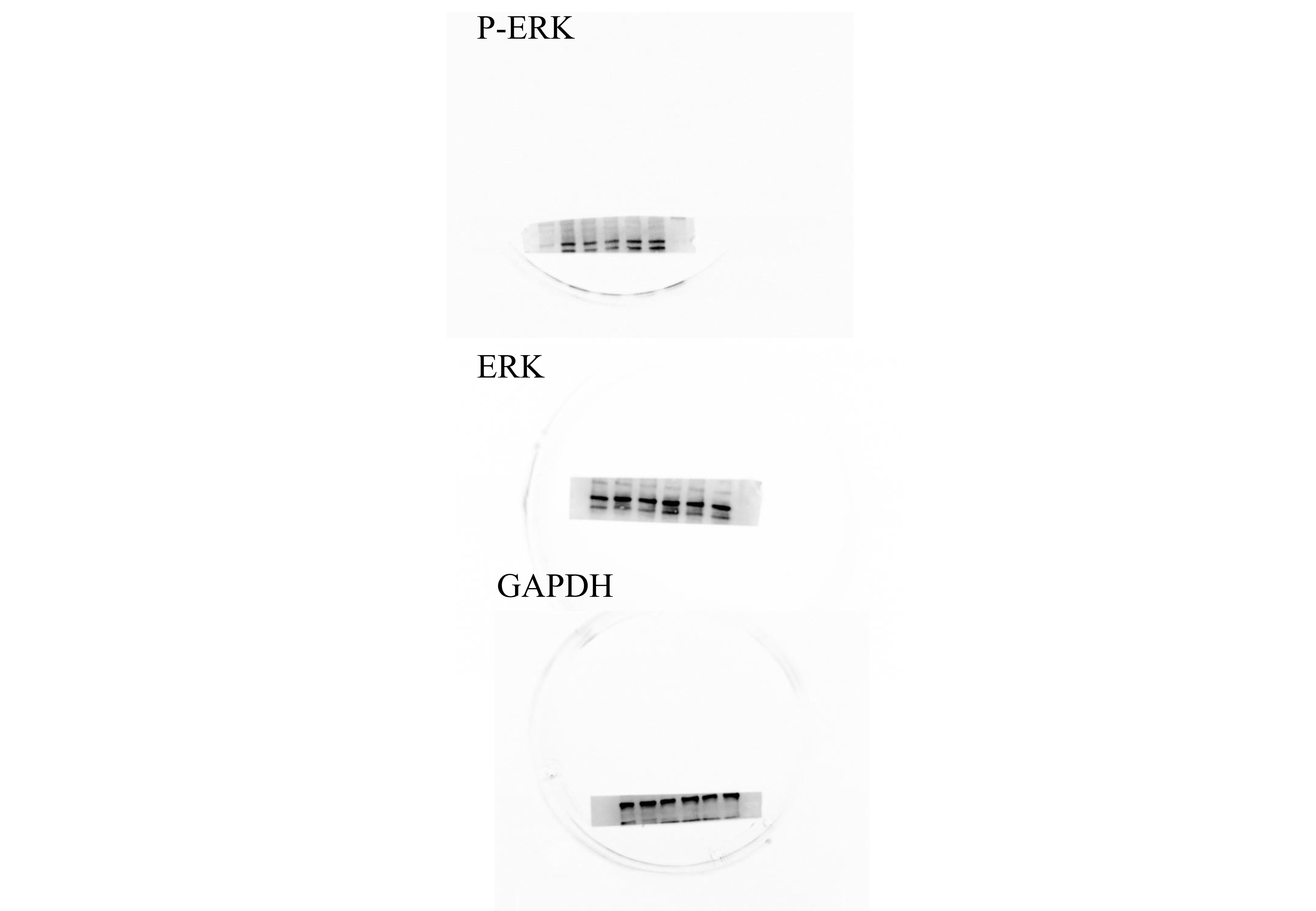

Supplement: Supplementary file 1 [file pharmaceuticals-19-00190-s001.zip › Supplementary Material S6-Western blot results/ERK-2.tif]

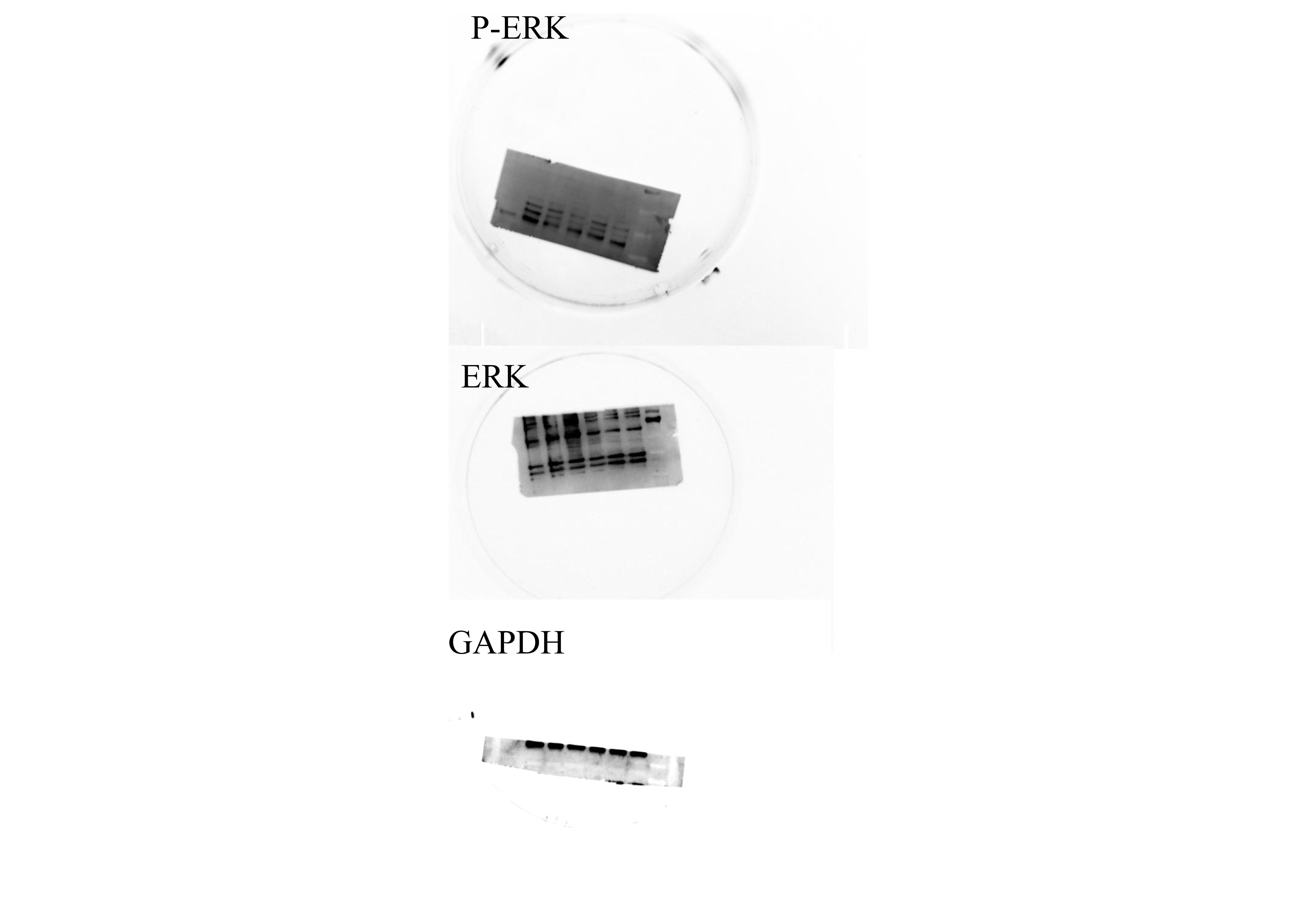

Supplement: Supplementary file 1 [file pharmaceuticals-19-00190-s001.zip › Supplementary Material S6-Western blot results/ERK-3.tif]

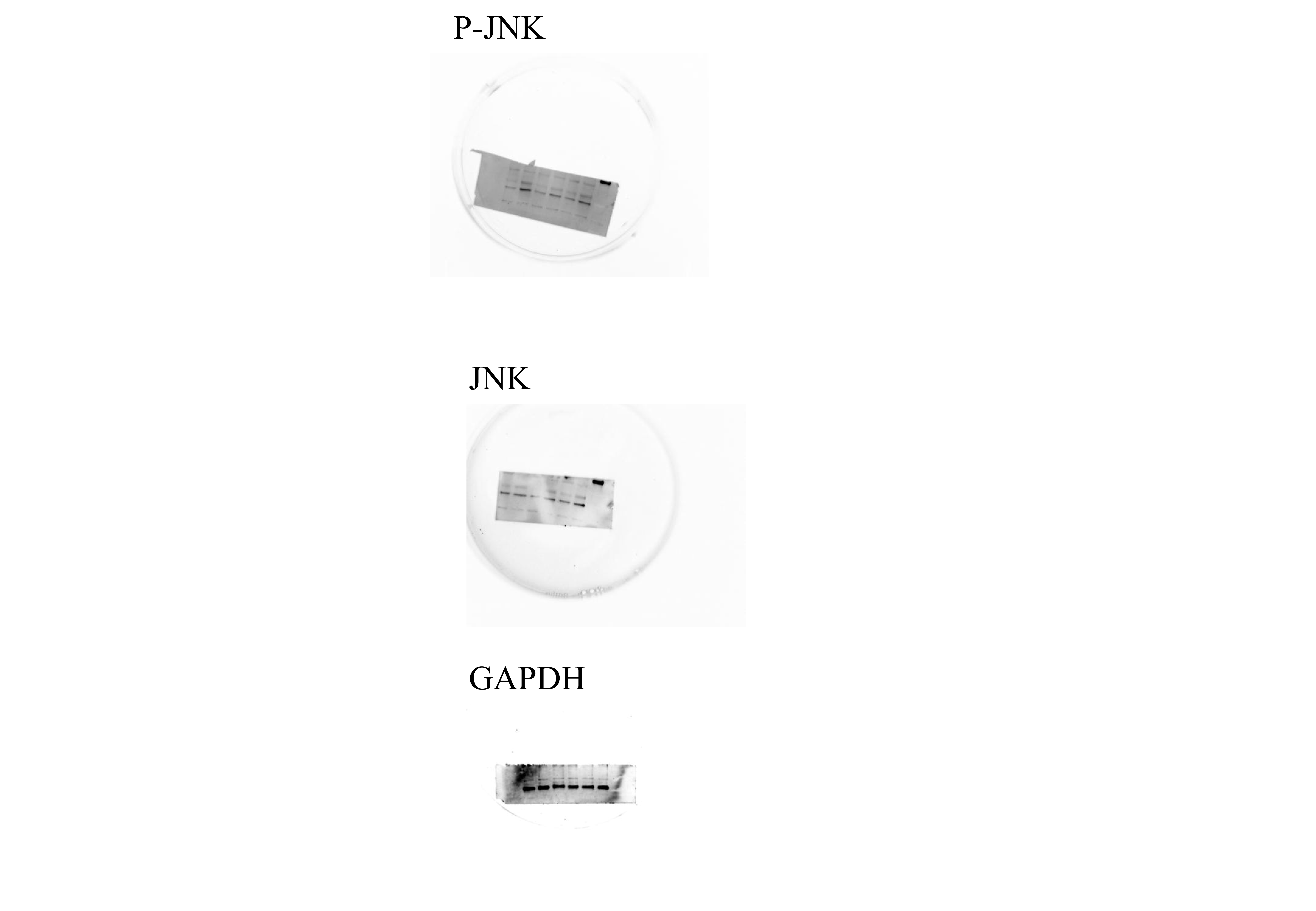

Supplement: Supplementary file 1 [file pharmaceuticals-19-00190-s001.zip › Supplementary Material S6-Western blot results/JNK-1.tif]

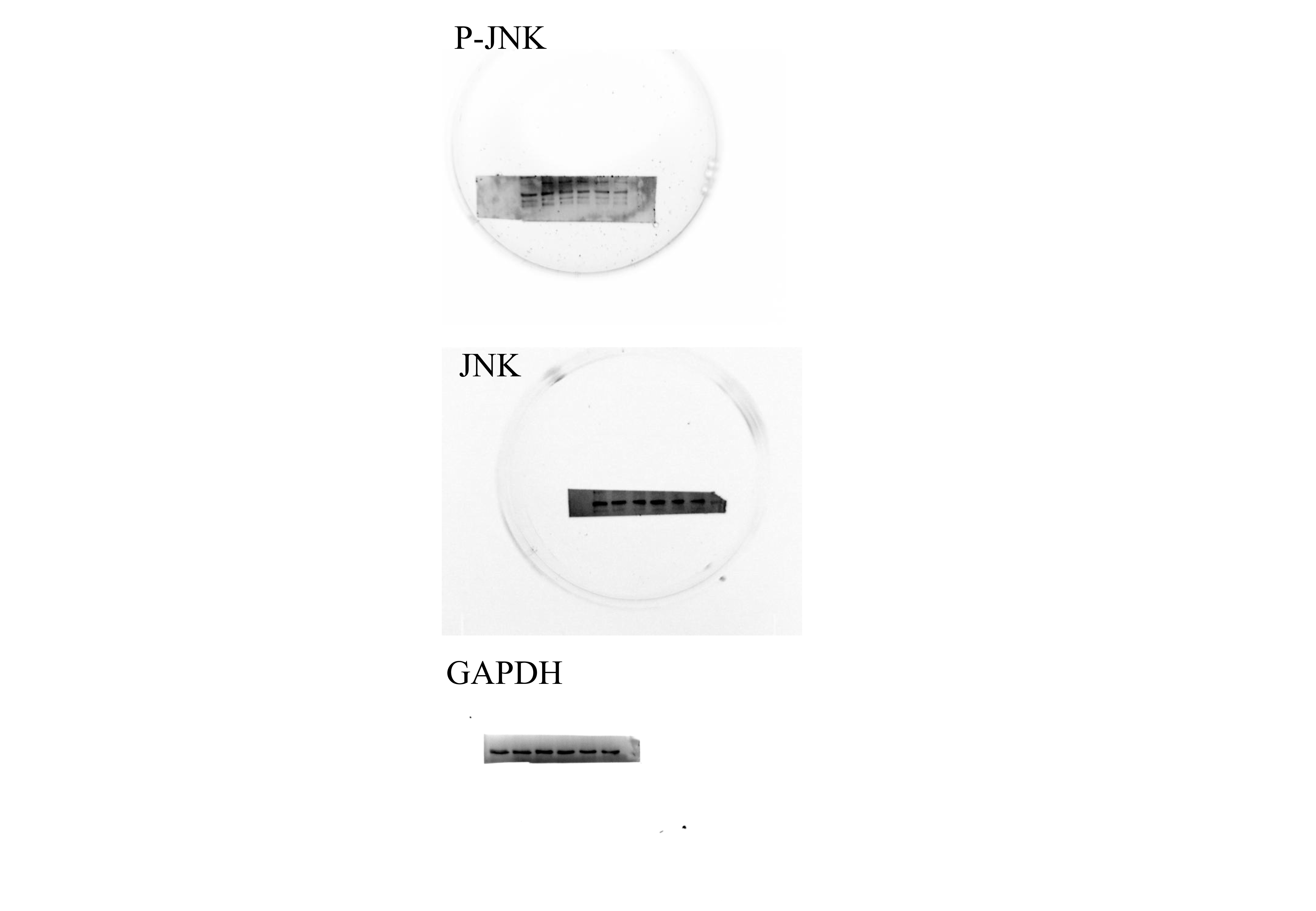

Supplement: Supplementary file 1 [file pharmaceuticals-19-00190-s001.zip › Supplementary Material S6-Western blot results/JNK-2.tif]

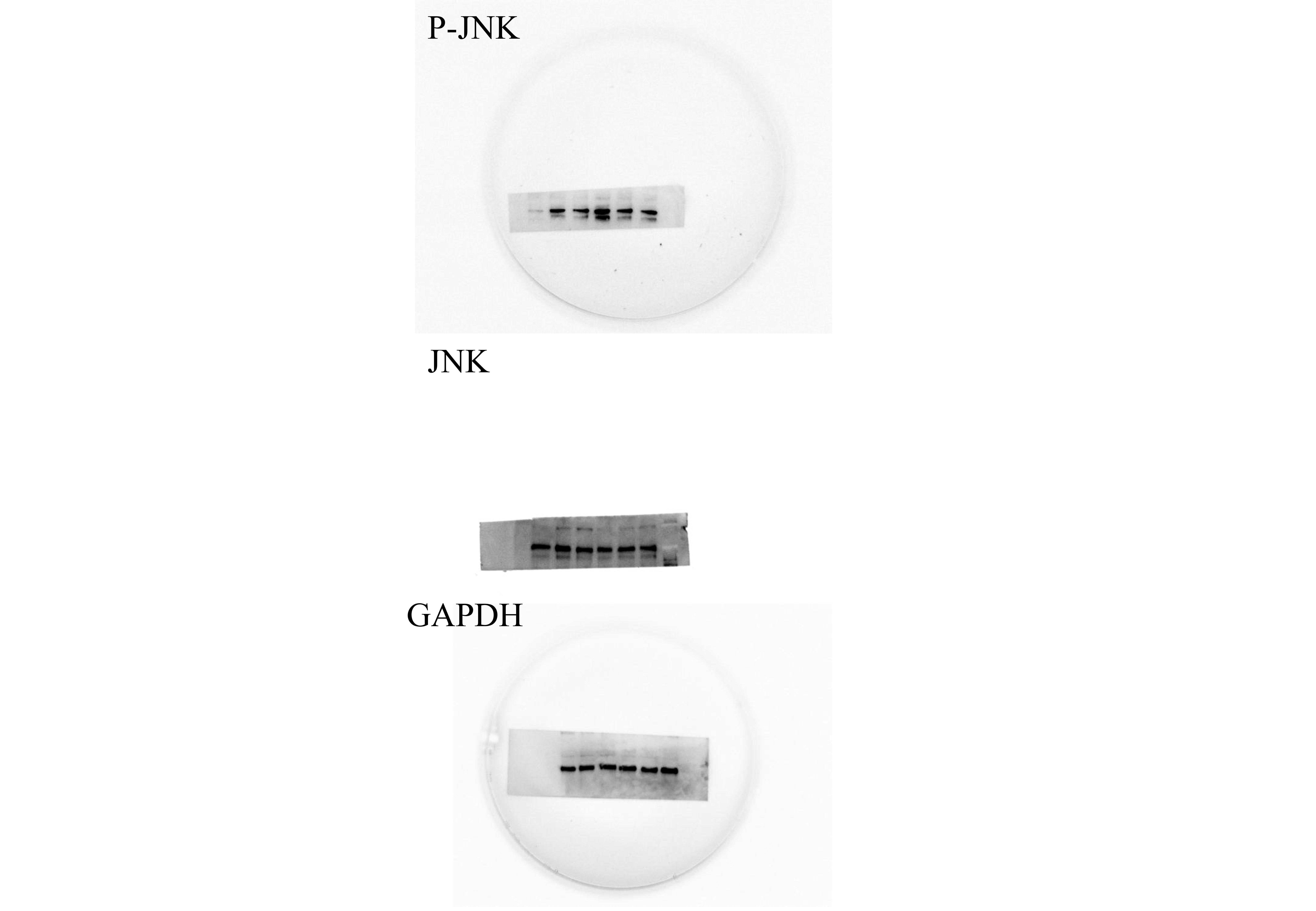

Supplement: Supplementary file 1 [file pharmaceuticals-19-00190-s001.zip › Supplementary Material S6-Western blot results/JNK-3.tif]

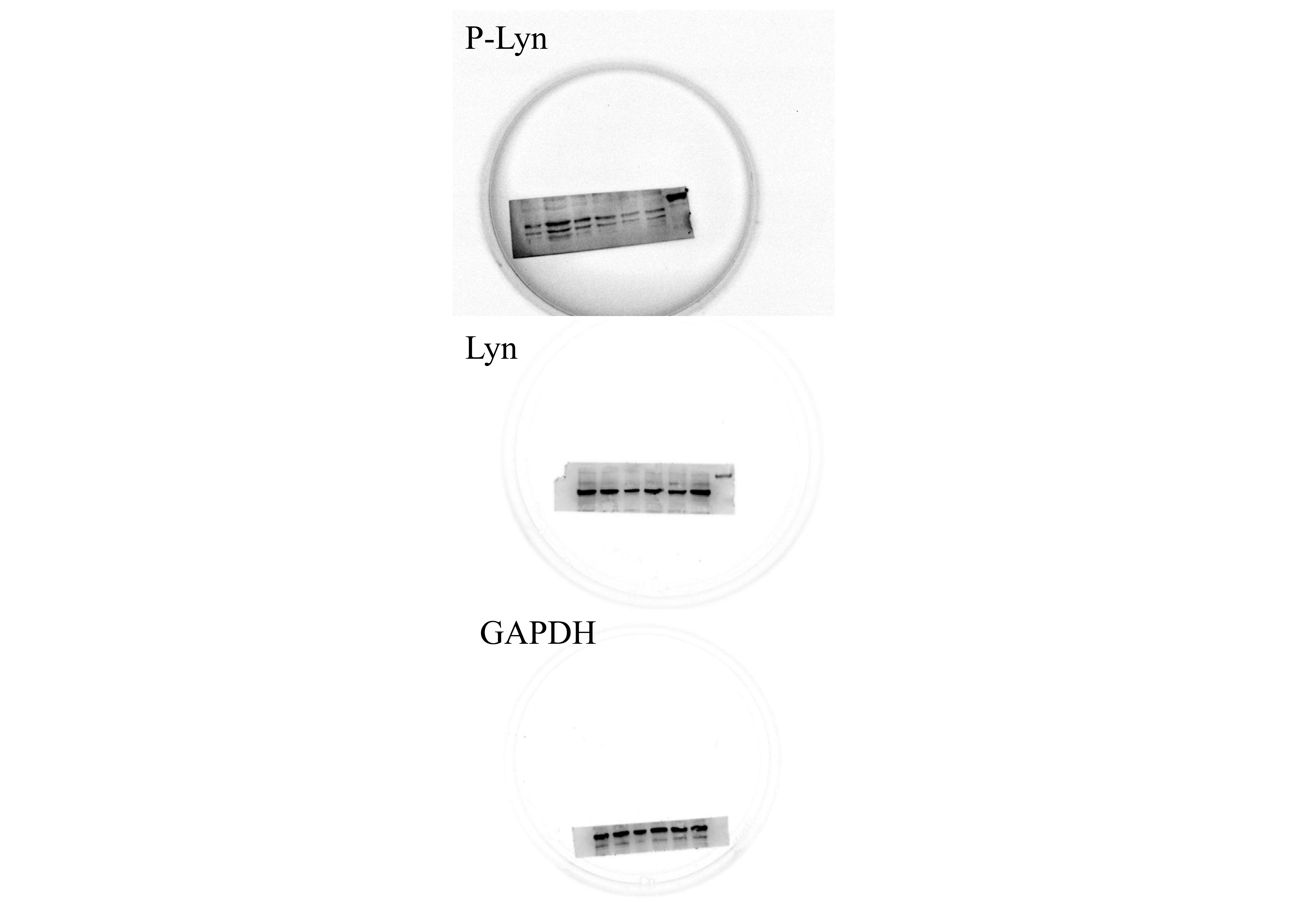

Supplement: Supplementary file 1 [file pharmaceuticals-19-00190-s001.zip › Supplementary Material S6-Western blot results/Lyn-1.tif]

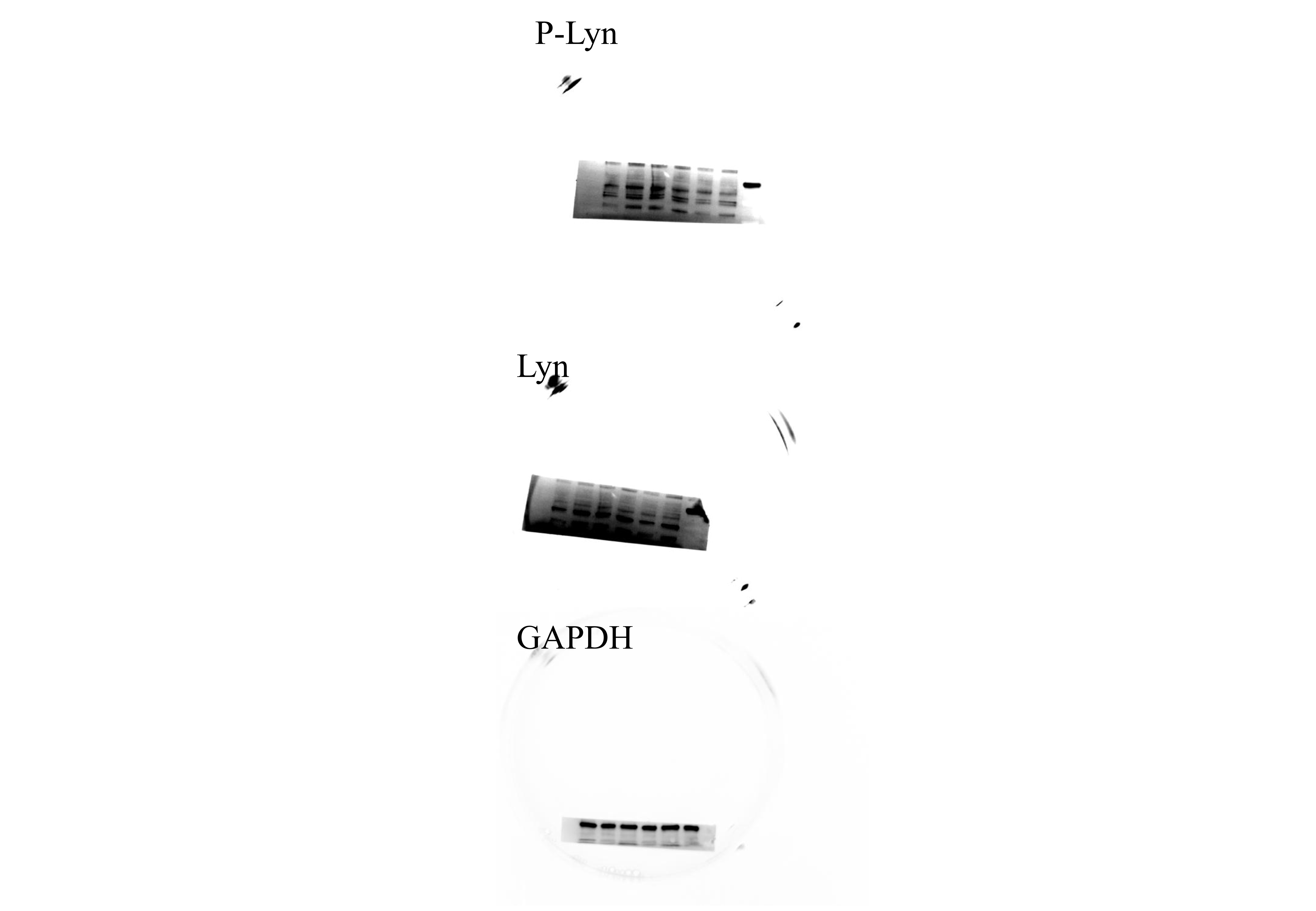

Supplement: Supplementary file 1 [file pharmaceuticals-19-00190-s001.zip › Supplementary Material S6-Western blot results/Lyn-2.tif]

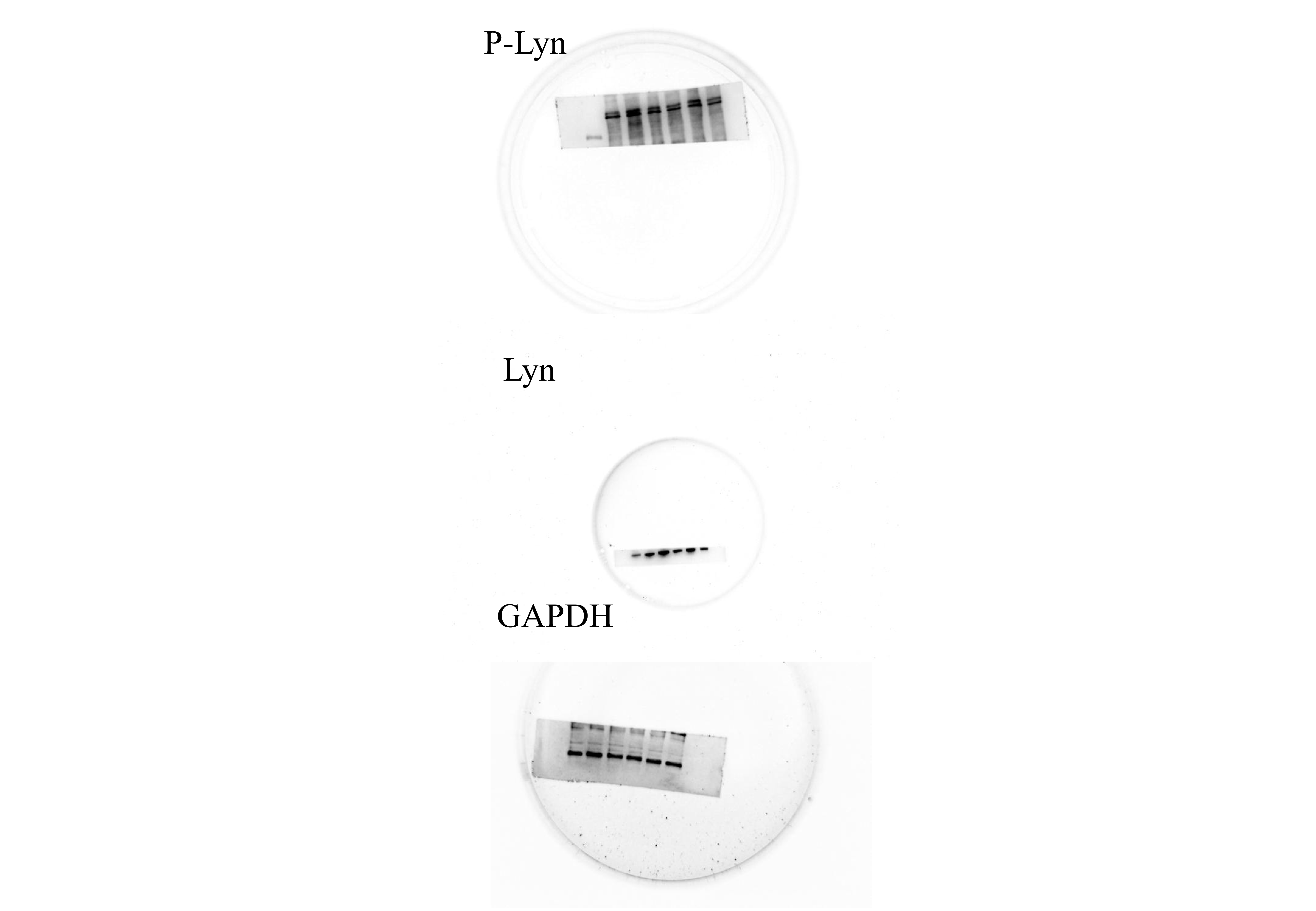

Supplement: Supplementary file 1 [file pharmaceuticals-19-00190-s001.zip › Supplementary Material S6-Western blot results/Lyn-3.tif]

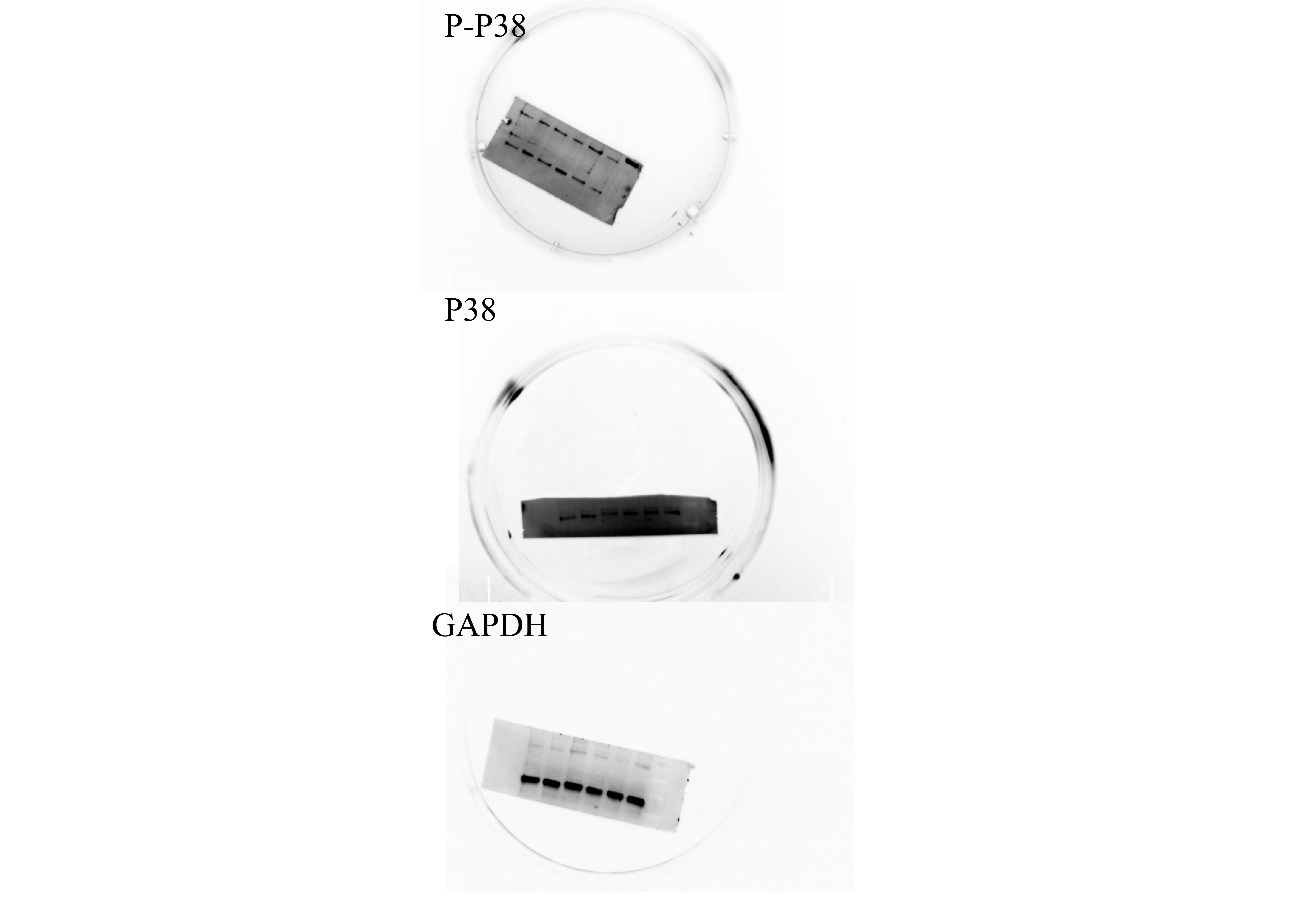

Supplement: Supplementary file 1 [file pharmaceuticals-19-00190-s001.zip › Supplementary Material S6-Western blot results/P38-1.tif]

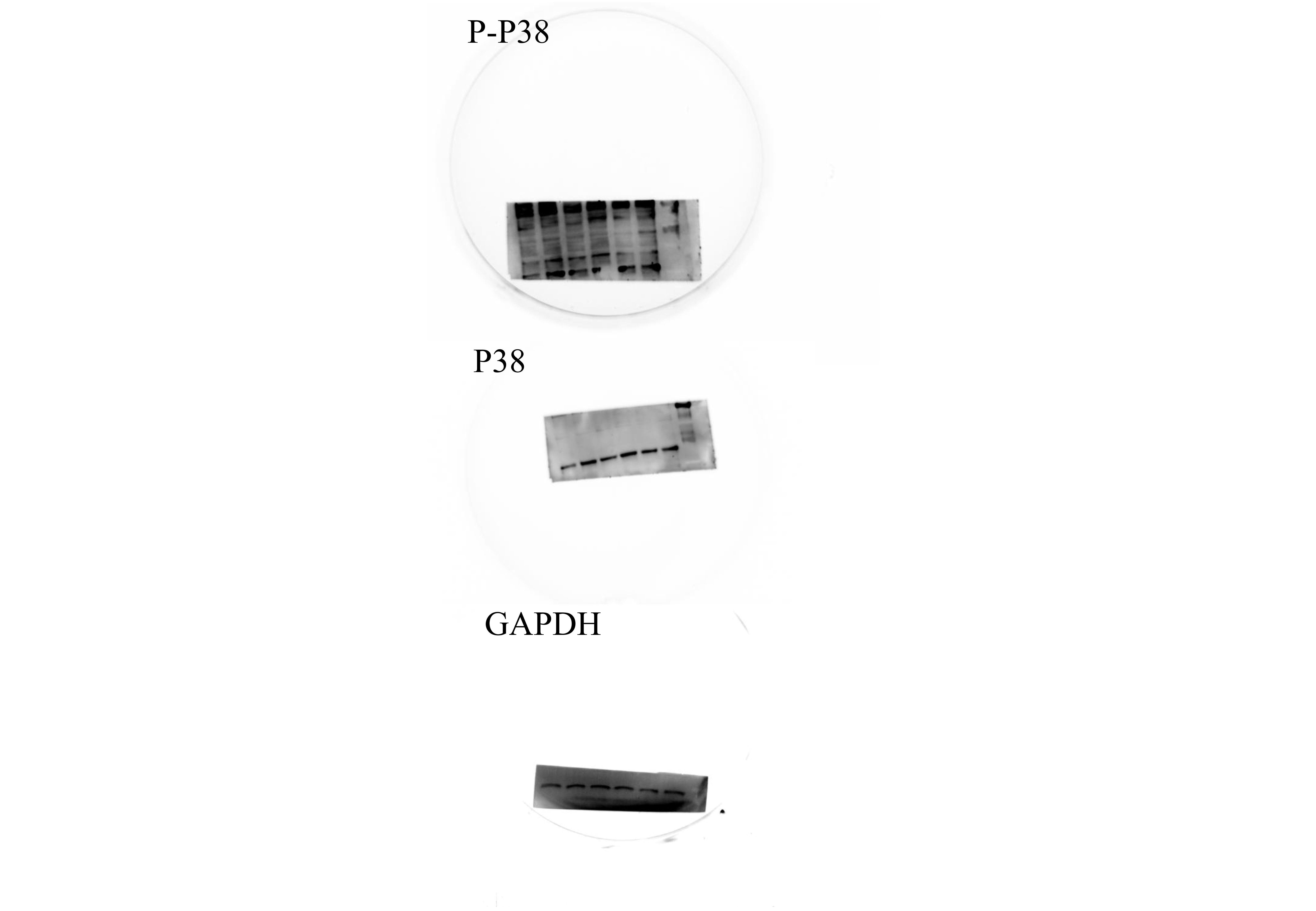

Supplement: Supplementary file 1 [file pharmaceuticals-19-00190-s001.zip › Supplementary Material S6-Western blot results/P38-2.tif]

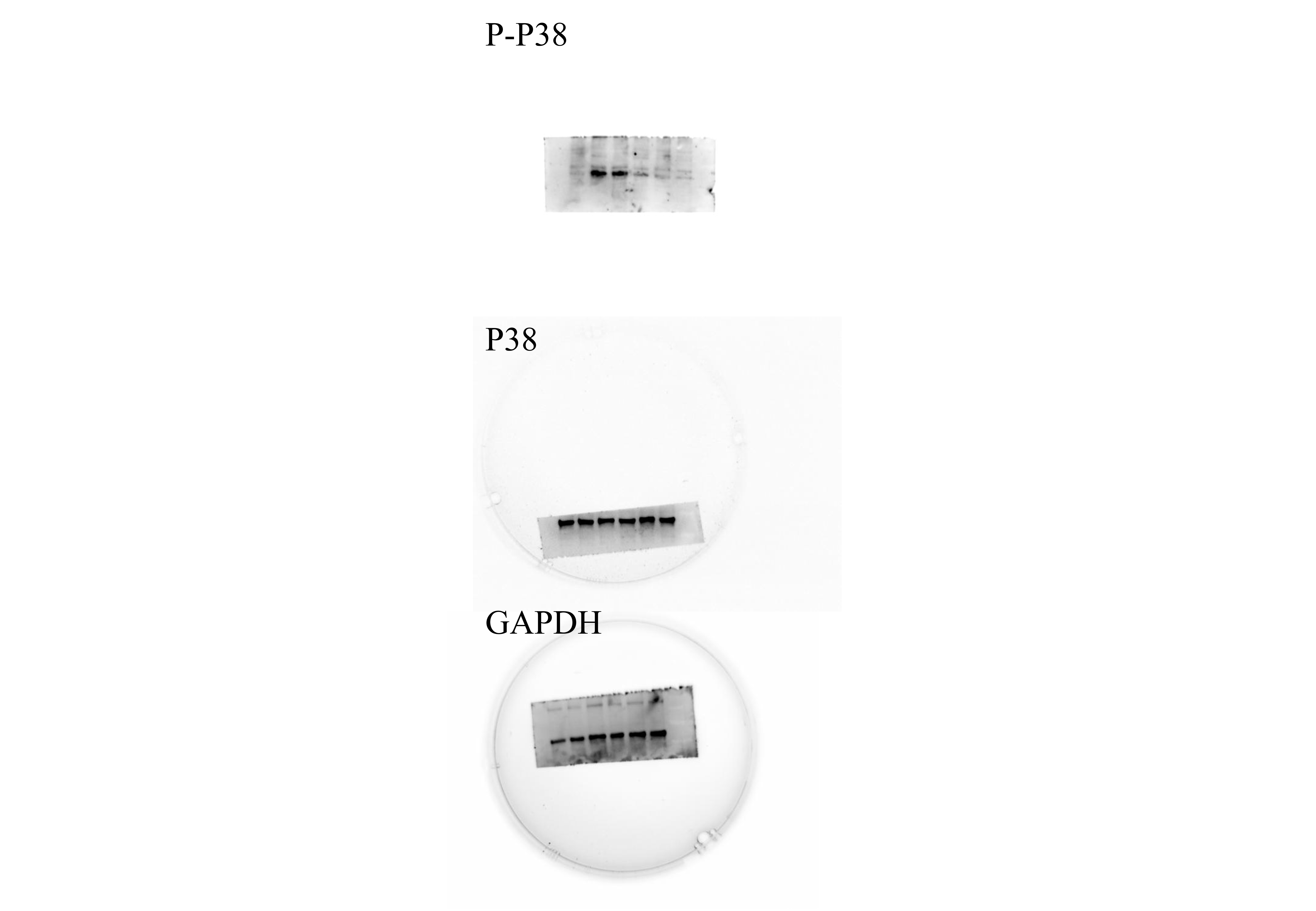

Supplement: Supplementary file 1 [file pharmaceuticals-19-00190-s001.zip › Supplementary Material S6-Western blot results/P38-3.tif]

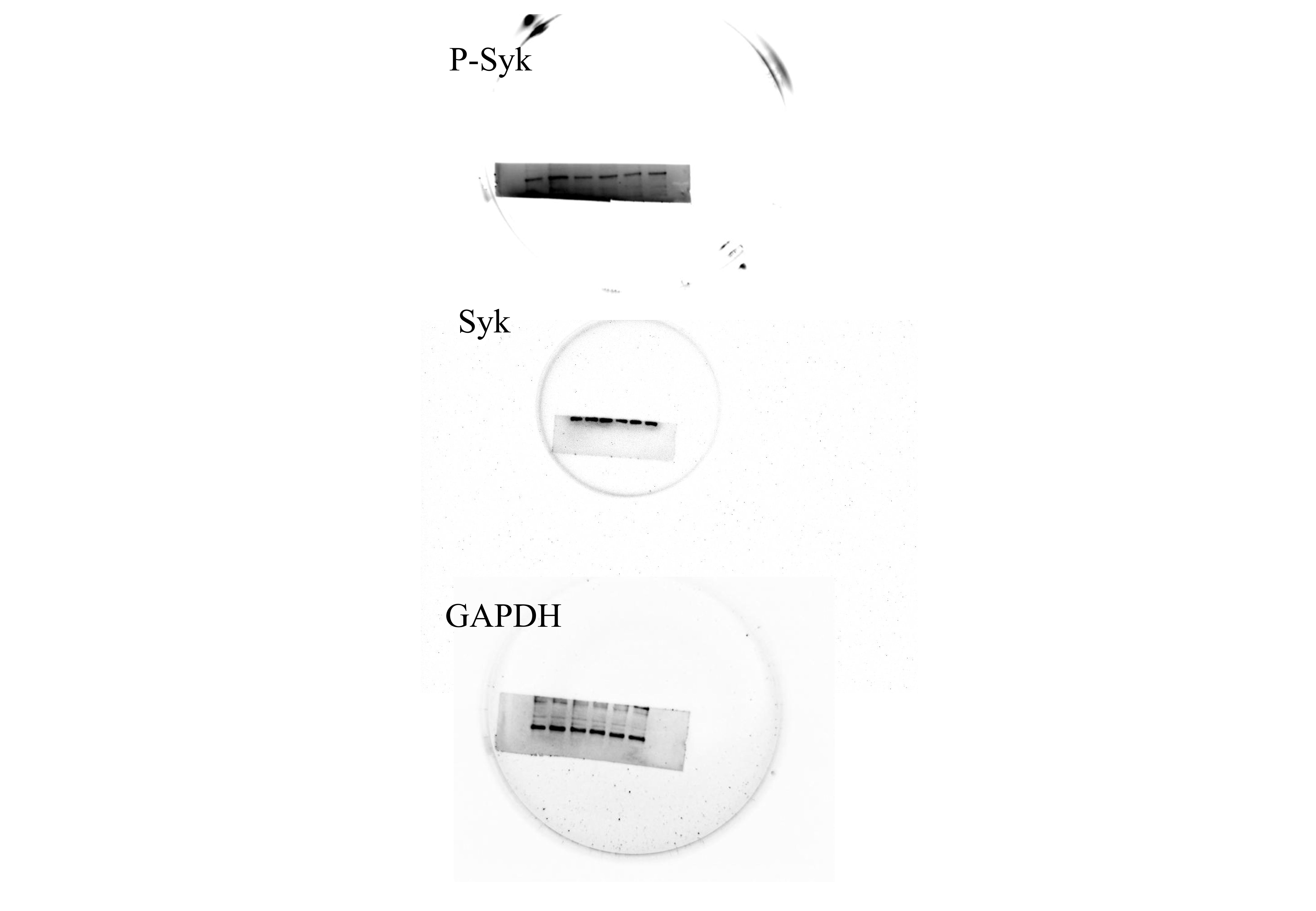

Supplement: Supplementary file 1 [file pharmaceuticals-19-00190-s001.zip › Supplementary Material S6-Western blot results/Syk-1.tif]

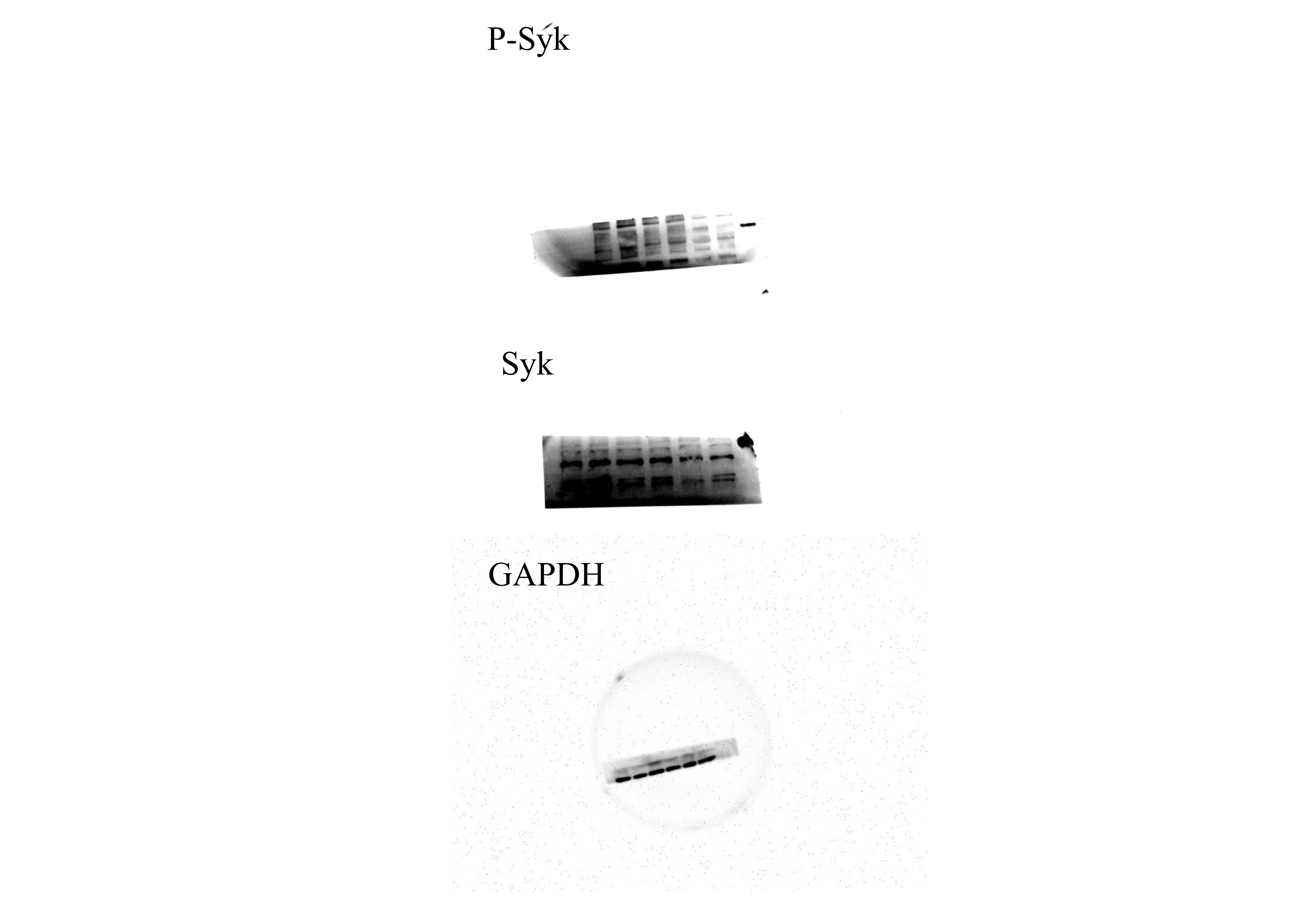

Supplement: Supplementary file 1 [file pharmaceuticals-19-00190-s001.zip › Supplementary Material S6-Western blot results/Syk-2.tif]

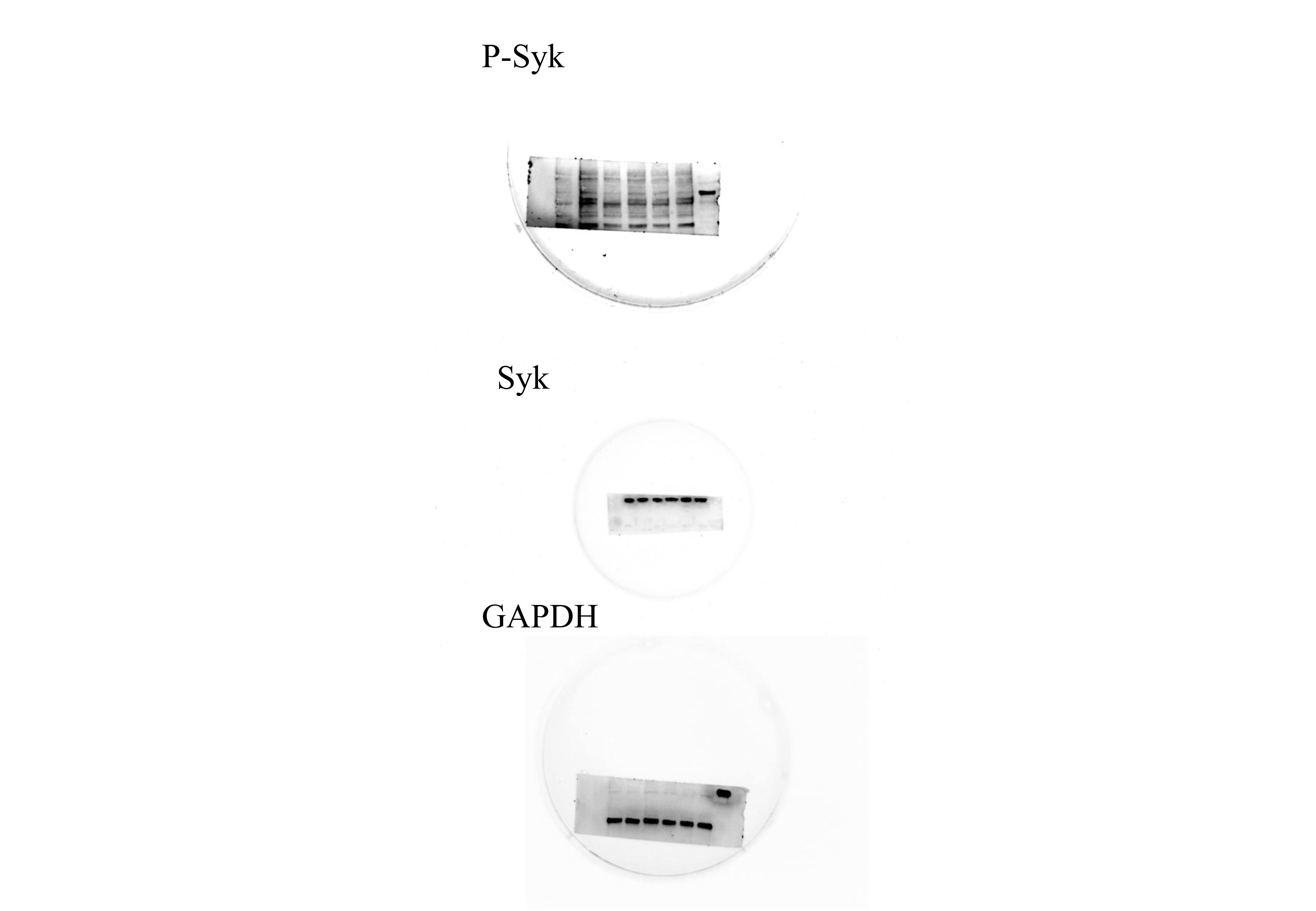

Supplement: Supplementary file 1 [file pharmaceuticals-19-00190-s001.zip › Supplementary Material S6-Western blot results/Syk-3.tif]
